# Comprehensive characterization of erythroid-specific enhancers in the genomic regions of human Krüppel-like factors

**DOI:** 10.1186/1471-2164-14-587

**Published:** 2013-08-28

**Authors:** Qian Xiong, Zhaojun Zhang, Kai-Hsin Chang, Hongzhu Qu, Hai Wang, Heyuan Qi, Yajuan Li, Xiuyan Ruan, Yaran Yang, Yadong Yang, Yanming Li, Richard Sandstrom, Peter J Sabo, Qiliang Li, George Stamatoyannopoulos, John A Stamatoyannopoulos, Xiangdong Fang

**Affiliations:** 1 CAS Key Laboratory of Genome Sciences and Information, Beijing Institute of Genomics, Chinese Academy of Sciences, Beijing 100101, P.R. China; 2 University of Chinese Academy of Sciences, Beijing 100049, P.R. China; 3 Division of Hematology, Department of Medicine, University of Washington, Seattle, WA 98195, USA; 4 Department of Genome Sciences, University of Washington, Seattle, WA 98195, USA; 5 Division of Medical Genetics, Department of Medicine, University of Washington, Seattle, WA 98195, USA

**Keywords:** DHS profiling, High-throughput sequencing, Erythroid, Enhancer, Krüppel-like factors

## Abstract

**Background:**

Mapping of DNase I hypersensitive sites (DHSs) is a powerful tool to experimentally identify *cis*-regulatory elements (CREs). Among CREs, enhancers are abundant and predominantly act in driving cell-specific gene expression. Krüppel-like factors (KLFs) are a family of eukaryotic transcription factors. Several KLFs have been demonstrated to play important roles in hematopoiesis. However, transcriptional regulation of KLFs via CREs, particularly enhancers, in erythroid cells has been poorly understood.

**Results:**

In this study, 23 erythroid-specific or putative erythroid-specific DHSs were identified by DNase-seq in the genomic regions of 17 human KLFs, and their enhancer activities were evaluated using dual-luciferase reporter (DLR) assay. Of the 23 erythroid-specific DHSs, the enhancer activities of 15 DHSs were comparable to that of the classical enhancer HS2 in driving minimal promoter (minP). Fifteen DHSs, some overlapping those that increased minP activities, acted as enhancers when driving the corresponding KLF promoters (KLF-Ps) in erythroid cells; of these, 10 DHSs were finally characterized as erythroid-specific KLF enhancers. These 10 erythroid-specific KLF enhancers were further confirmed using chromatin immunoprecipitation coupled to sequencing (ChIP-seq) data-based bioinformatic and biochemical analyses.

**Conclusion:**

Our present findings provide a feasible strategy to extensively identify gene- and cell-specific enhancers from DHSs obtained by high-throughput sequencing, which will help reveal the transcriptional regulation and biological functions of genes in some specific cells.

## Background

Biological processes such as proliferation, apoptosis, differentiation, development, and aging require elaborately orchestrated spatial and temporal gene expression, which are often under the control of *cis*-regulatory elements (CREs). CREs, including promoters, enhancers, silencers, insulators and locus control regions (LCRs) etc., are abundant in the human genome [1]. Characterization of CREs in the genome contributes to understand the complexities of gene transcription and expression in different biological systems [2,3]. In the past decade, the project ENCyclopedia of DNA Elements (ENCODE) has facilitated the prediction of functional elements including CREs in the human genome [4]. However, CRE characterization in terms of gene and cell specificities as well as chromatin context dependency remains a huge challenge.

Nucleosome-depleted DNA regions, characterized by their sensitivity to nuclease digestion, are closely associated with almost all known classes of active CREs. In contrast, DNA regions tightly wrapped in nucleosomes and higher-order structures are more resistant to nuclease digestion. Therefore, DNase I hypersensitive sites (DHSs) mark many types of CREs [2,5]. Individual DHSs within small regions of the genome (10–20 kb) have been traditionally identified using Southern blot analysis [6]. However, this labor-intensive and low-throughput approach cannot be scaled to study large chromosomal regions and entire genomes. By hybridizing DNase I-digested fragments to tiled microarrays, the DNase-chip assay provides an undirected, unbiased, highly sensitive and specific strategy to simultaneously identify thousands of DHSs within any region of interest or even the entire genome, with a resolution of 200–500 bases [7]. Furthermore, DNase-seq (identification of DNase I-digested fragments by next-generation sequencing) allows genome-wide mapping of DHSs with base-pair resolution [2]. Using these high-throughput technologies, DHS mapping is emerging as a powerful tool for locating open chromatin regions that encompassing many types of CREs within the genome [2,3,8] and thus it facilitates the delineation of the roles of DHSs in regulating tissue- and developmental stage-specific expression of nearby genes [9,10].

Enhancers are the most variable CREs that can regulate the expression of genes from a long distance and in a position- and orientation-independent manner [11]. In general, it is accepted that enhancers function by first recruiting sequence-specific transcription factors (TFs) that recognize short DNA motifs within the enhancers. Upon binding to enhancers, the sequence-specific TFs recruit mediator complexes, histone modifiers and chromatin remodelers to activate the transcription of target genes [12]. Enhancers often exist in a cell- and developmental stage-specific manner [13], and the distribution of cell-specific enhancers correlates well with cell-specific gene expression [14], suggesting that they are the primary force driving spatial- or temporal-specific gene expression. To date, several lines of evidences have demonstrated the roles of erythroid-specific enhancers in driving erythroid-specific gene expression. LCR at the β-globin locus is the most prominent erythroid enhancer that exerts a strong effect specifically on erythroid cells. This LCR enhances the developmental stage-specific expression of globin genes and the expression of linked heterogeneous non-globin genes in erythroid cells by interacting with respective promoters [15]. HS2, a classical enhancer located in LCR, appears to be functional in erythroid cells at both embryonic and adult developmental stages, suggesting its crucial roles in the activation of globin genes in erythroid cells throughout ontogenesis [16]. Other erythroid-specific enhancers have also been found in the genomic regions of *GATA–1,* stem cell leukemia (*SCL*), L-type pyruvate kinase and 5-aminolevulinate synthase 2 (*ALAS2*) genes [17-20], which may contribute to the restricted expression of these genes in the erythroid lineage. A strikingly large number of enhancers have been systematically identified in erythroid K562 cells using chromatin immunoprecipitation followed by genome tiling array (ChIP-chip) analysis [14]. The characterization of these and other erythroid or erythroid-specific enhancers in the human genome will facilitate the understanding of regulation and functions of associated genes in erythroid cells.

Krüppel-like factors (KLFs) are a subfamily of zinc-finger proteins that contain three tandem Cys_2_His_2_ zinc fingers at the highly conserved carboxyl terminus. KLFs are important components of the eukaryotic cellular transcriptional machinery. By regulating the expression of several genes driven by GC-rich or CACCC-containing promoters, KLFs participate in many biological processes, including hematopoiesis, adipogenesis, stem cell maintenance, and tumorigenesis [21]. In particular, several KLFs have been demonstrated to play crucial roles in erythroid differentiation. Globin genes, including α-, ϵ-, γ- and β-globin genes, are prominent biomarkers in erythroid cells, and their spatial and temporal expression is closely correlated with erythroid differentiation and development [22]. KLF1 (EKLF), an erythroid-specific TF, activates adult β-globin gene expression [23] and regulates gene switching from γ- to β-globin [24] as well as definitive hematopoiesis. Other KLFs mainly play significant roles in primitive hematopoiesis. For example, KLF4 activates the expression of α- and γ-globin genes [25,26]. Expression of embryonic ϵ- and fetal γ-globin genes is stimulated by KLF2 [27], KLF11 [28,29], and KLF13 [30] but is suppressed by KLF3 [31] and KLF8 [32]. In addition, KLF6 [33] and KLF17 [34] are required for primitive hematopoiesis. Hematopoietic defects or anemia have been observed in several *Klf*-knockout mice, including mice lacing *Klf1*[35], *Klf2*[27], *Klf3*[36], *Klf6*[33], and *Klf13*[37]. Interestingly, cross-regulation among KLFs has been reported during erythropoiesis, erythroid differentiation, and globin gene regulation [36,38,39]. However, till date, few studies have been conducted to investigate the *cis-*transcriptional regulation of KLFs by erythroid-specific enhancers, with the exception of the study on murine KLF1 enhancers [40]. Therefore, characterization of erythroid-specific enhancers will shed light on molecular mechanisms that regulate transcription, expression, and functions of KLFs in erythroid cells.

Here, we characterized gene- and cell-specific enhancers in the genomic regions of human KLFs extensively by combining high-throughput sequencing data as well as biochemical and bioinformatic analyses. Our mRNA-seq data in human embryonic stem cells (HESC) and three primary erythroid cell types demonstrated that human KLFs, including KLF1, KLF3, KLF6, KLF9, KLF10, KLF11, KLF13, and KLF16, were up-regulated in erythroid cells as compared to HESC. We also mapped DHSs in the genomic regions of 17 human KLFs across four erythroid and seven non-erythroid cell types and screened out 23 erythroid-specific or putative erythroid-specific DHSs. Using the dual-luciferase reporter (DLR) assay, we identified 10 (43%) erythroid-specific enhancers embedded in the genomic regions of KLF1, KLF6, KLF9, and KLF13. The nature of these identified erythroid-specific enhancers was confirmed by a series of bioinformatic and biochemical analyses, contributing to understand the mechanism by which KLFs are regulated in erythroid cells. Our present findings provide a feasible strategy to characterize cell- and gene-specific enhancers from DHSs generated from high-throughput sequencing across various cell types, and to facilitate the illustration of transcriptional regulation and functions of genes in specific cell types.

## Results

### Expression of some KLFs is up-regulated in erythroid cells

**Figure 1:**
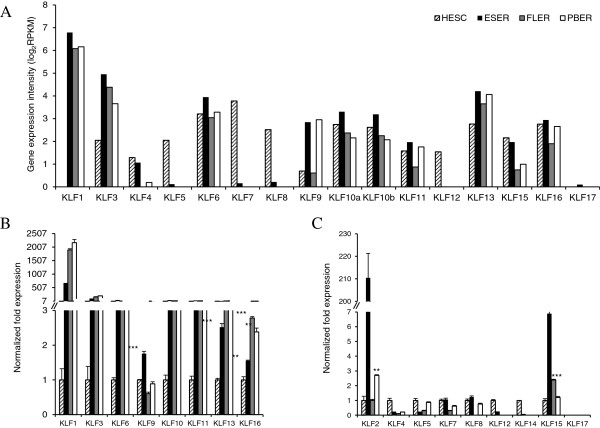
**Differential expression patterns of KLFs in HESC and primary erythroid cells. (A)** Gene expression profile of KLF1-17 in undifferentiated HESC and three primary erythroid cells (ESER, FLER, and PBER) examined using mRNA-seq analysis. Gene expression intensity was calculated by normalizing the read counts to reads per kilobase of the exon model per million mapped reads (RPKM) according to the gene length and total mapped reads. KLF2 and KLF14 were not detected and thus were not shown in this figure, whereas KLF10a and 10b represented two KLF10 isoforms. **(B)** Gene expression profile of KLF1, 3, 6, 9, 10, 11, 13, and 16 in undifferentiated HESC and three primary erythroid cells examined using quantitative real-time PCR. Transcript levels of KLFs were calculated in relation to that of 18S ribosomal RNA, and the expression levels of KLFs in ESER, FLER, and PBER were normalized to those in HESC. The error bars above each column indicate standard error of the mean (SEM) between triplicates. The Y-axis breaks at 3. Asterisks indicate that the differences between the levels of individual transcripts in erythroid cells (with Y values ranging from 1 to 3) and those in HESC were statistically significant by independent-samples *t*-test, ***p < 0.001, **p < 0.005. **(C)** Gene expression profile of KLF2, 4, 5, 7, 8, 12, 14, 15, and 17 in undifferentiated HESC and three primary erythroid cells examined using quantitative real-time PCR.

Expression patterns of KLFs were obtained from the mRNA-seq dataset that was originally designed to explore the dynamic transcriptomes during human erythroid differentiation and development (Yang Y, Wang H, Chang KH, Qu H, Zhang Z, Xiong Q, Qi H, Cui P, Lin Q, Ruan X, *et al*: Transcriptome dynamics during human erythroid differentiation and development, submitted). The following cell types were examined: undifferentiated HESC, embryonic stem cells-derived erythroid cells (ESER), fetal liver-derived erythroid cells (FLER), and adult mobilized peripheral blood CD34+ cells-derived erythroid cells (PBER). As shown in Figure 1A, of the 17 KLFs examined, the expressions of KLF2 and KLF14 were not detected, whereas KLF17 was poorly expressed in ESER. The expressions of KLF4, KLF5, KLF7, KLF8, KLF12, and KLF15 in HESC were higher than those in erythroid cells, whereas those of the remaining eight KLFs were higher in erythroid cells than in HESC. The expressions of all the 17 KLFs were further evaluated using quantitative real-time PCR. With the exception of KLF2 and KLF15, the expression patterns of the remaining 15 KLFs measured by PCR correlated well with mRNA-seq results (Figure 1B, C). The cause of the inconsistency in KLF2 and KLF15 measurement is currently not clear but it could be platform related. Nevertheless, we proposed that the higher expressions of the eight KLFs detected by both two platforms may be attributed to the presence of erythroid-specific enhancers.

### DHSs in KLF genomic regions are distributed diversely in various cell types

**Figure 2:**
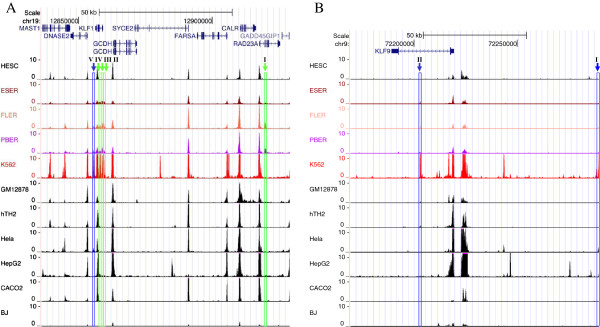
**Distribution of DHSs in the genomic regions of *KLF1 *and *KLF9 *genes.** Chromatin profiles for *KLF1***(A)** and *KLF9***(B)** are shown to illustrate the distribution of DHSs in the genomic regions of *KLF* genes in four erythroid (in color) and seven non-erythroid (in black) cell lines. Erythroid-specific or putative erythroid-specific DHSs were named with Roman numbers. Erythroid-specific DHSs, which were present only in human erythroid cells, were indicated by arrows and columns in green. Putative erythroid-specific DHSs, which were also present in non-erythroid cell types but at lower intensities, were indicated by arrows and columns in blue. Therefore, KLF1-I, II, III, and IV **(A)** were considered as erythroid-specific DHSs, of which KLF1-I was located in the intron region of the *RAD23A* gene within the defined genomic region, and KLF1-V was a putative erythroid-specific DHS because of its presence in HeLa cells. Similarly, upstream KLF9-I and intronic KLF9-II were considered as putative erythroid-specific sites **(B)**.

The DNase-seq dataset used in this study was generated by the University of Washington [41]. The dataset is composed of DNase-seq data in four erythroid cell types, including three primary erythroid cells (ESER, FLER, and PBER) and erythroleukemia K562 cells, to cover all the possible DHSs in the erythroid lineage and seven non-erythroid cell types, including HESC, GM12878, hTH2, HeLa, HepG2, CACO2, and BJ, to differentiate erythroid-specific DHSs from non-erythroid ones. A false discovery rate (FDR) threshold of 0.5% was used to define DHSs in each cell type. DHS mapping was profiled for all the *KLF* gene loci from 70 kb upstream of the transcription start sites (TSSs) to 20 kb downstream of the poly (A) sites. The current coverage of gene loci was determined based on the following reasons: First, the regions covering approximately 100 kb encompassed almost all intensive DHSs around the corresponding KLFs in the four cell types studied (Figure 2, Additional file 1: Figures S1 and S2). Second, CTCF binding is reported to mark boundary elements between neighboring genes [42]. These approximate 100-kb regions contain such ubiquitous CTCF-binding sites in the various cell types employed in the present study (data from UCSC Browser). The diverse distribution patterns of KLF DHSs among the various established cell types and HESC were shown in Figure 2, Additional file 1: Figures S1 and S2, coinciding with the varying expression levels of *KLF* genes in these cell types. DHSs were considered to be erythroid specific if they were only present in erythroid cells and were classified as putative erythroid specific if they were present in erythroid cells, while much subdued peaks were also detected in one or two non-erythroid cell types.

Figure 2A illustrated the DHS profiling of KLF1. Five prominent DHSs were detected in the *KLF1* genomic region; of these KLF1-I was located at > 60 kb upstream of the *KLF1* gene and was only present in three primary erythroid cells, whereas KLF1-II, III, IV, and V were located proximal to the *KLF1* gene and were present in both primary erythroid cells and erythroleukemia K562 cells. KLF1-V was a putative erythroid-specific site because a small peak for this site was also present in non-erythroid HeLa cells. The diverse cellular presentation of DHSs was also observed in the profile of *KLF9* (Figure 2B), with two putative erythroid-specific DHSs—KLF9-I and KLF9-II—located 70 kb upstream or in the intron of the gene respectively.

Other erythroid-specific or putative erythroid-specific sites in the *KLF* profiles are shown in Additional file 1: Figure S1. Erythroid-specific DHSs include DHS-I of *KLF2* (Additional file 1: Figure S1A), DHS-I and II of *KLF3* (Additional file 1: Figure S1B), DHS-III and IV of *KLF6* (Additional file 1: Figure S1C), DHS-I of *KLF10* (Additional file 1: Figure S1D), DHS-I and II of *KLF13* (Additional file 1: Figure S1F), DHS-II of *KLF16* (Additional file 1: Figure S1G), and DHS-I of *KLF17* (Additional file 1: Figure S1H). In addition, DHS-III of *KLF3* (Additional file 1: Figure S1B), DHS-I and II of *KLF6* (Additional file 1: Figure S1C), DHS-I of *KLF11* (Additional file 1: Figure S1E), DHS-III of *KLF13* (Additional file 1: Figure S1F), and DHS-I of *KLF16* (Additional file 1: Figure S1G) were identified as putative erythroid-specific DHSs. The features of all the 23 erythroid-specific or putative erythroid-specific DHSs located in the *KLF* gene loci are summarized in Additional file 2: Table S1. DHS profiles of KLFs without erythroid-specific or putative erythroid-specific DHSs are shown in Additional file 1: Figure S2. It is also of interest to note that while KLF4 has been employed as a major reprogramming factor required to reverse the highly differentiated somatic cells into pluripotent cells [43], its expression in HESC was lower than that of many other KLFs, consistent with ENCODE/Caltech RNA-seq data available on UCSC Browser. As shown in Additional file 1: Figure S2A, weak peaks of DHSs (in HESC, ESER, FLER, and PBER) were found to be dispersed in the *KLF4* locus, which could account for its relatively lower expression than that of the other family members in the present and previous studies [44] and its dispensable role for the self-renewal and pluripotency of ES cells [43,45]. In particular, DHS peaks in the KLF4 promoter region tend to decrease during erythroid differentiation, which may explain the down-regulation of KLF4 expression in erythroid cells compared with that in HESC (Figure 1).

Among the 23 prominent erythroid-specific or putative erythroid-specific DHSs, 18 (78%) were located upstream of TSSs or downstream of poly (A) sites of the *KLF* genes. Only four (17%) DHSs were proximal (< 2 kb) to TSSs, whereas 15 (65%) were distal (> 10 kb) to TSSs (Additional file 1: Figure S3A). Our data on the identified erythroid-specific DHSs are comparable to those of a previous DNase-chip report on the distribution of cell type-specific DHSs within 1% of the human genome from six diverse cell types [3]. In contrast, with the exception of the erythroid-specific DHSs in the *KLF2* and *KLF17* regions (Additional file 1: Figure S1A, H), most erythroid-specific or putative erythroid-specific DHSs were present in the eight *KLF* genes that were up-regulated in erythroid cells (Figure 1, Figure 2, Additional file 1: Figure S1B-G). Moreover, erythroid-specific or putative erythroid-specific DHSs were absent in the genomic regions of several *KLF* genes (Additional file 1: Figure S2), which did not have increased expression in erythroid cells (Figure 1), implying that these KLFs may not function in erythroid cells or that they were not activated by erythroid-specific enhancers.

### Approximately 65% erythroid-specific or putative erythroid-specific DHSs are enhancers

**Figure 3:**
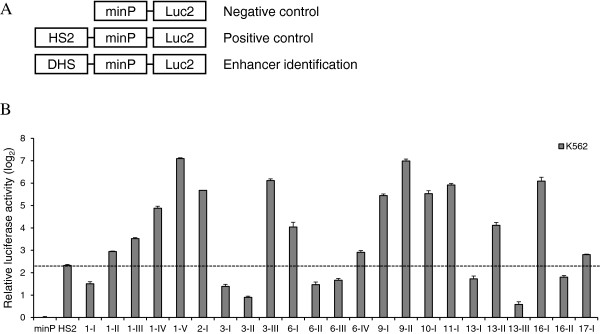
**Enhancer identification in K562 cells using the DLR assay with minP. (A)** The constructs used in the reporter assay system are depicted. The pGL4.23 vector containing one minP was set as a negative control. The pGL4.23 construct with enhancer HS2 was set as a positive control. pGL4.23 constructs with DHSs of interest in the place of HS2 were used to evaluate the enhancer activities of DHSs. **(B)** Enhancer activities evaluation of 23 erythroid-specific or putative erythroid-specific DHSs in K562 cells. Each construct was transfected in triplicate at a time and transfections were repeated at least twice. For each construct, the firefly luciferase activity was normalized to that of *Renilla* luciferase. The relative luciferase activity was shown in log_2_ scale, with that of minP set to 0. Standard deviations were shown as error bars above each column. DHSs with 5-fold or higher activities than that of minP were defined as enhancers.

Cell type-specific DHSs have been reported to act as enhancers [3]. To identify which erythroid-specific DHSs can serve as enhancers, DLR assay was performed to evaluate the enhancer activity of 23 DHSs in driving TATA box-containing minimal promoter (minP) [46]. K562 cells are immature erythroid cells widely used in studies of erythroid differentiation or other functions of the erythroid lineage; K562 is also one of the tier 1 cell types used in the ENCODE project with massive data available for subsequent analyses. Therefore, we selected the K562 cells to identify erythroid-specific enhancers *in vitro*. We found that enhancer HS2 in human β-globin LCR strongly activated minP in erythroid K562 cells (Figure 3). Therefore, HS2 was chosen as the positive control for enhancer activity evaluation in this assay as previous reports did [15,16,47]. HS2 activated minP by approximately 5 fold in this study. Therefore, we defined DHSs that could activate minP by 5 fold, which was 2.32 after log_2_ transformation as presented in Figure 3, or higher as enhancers. We found that 15 (65%) erythroid-specific or putative erythroid-specific DHSs had enhancer activity, with the activity of some being much stronger than that of HS2 (Figure 3), suggesting that minP was sensitive enough and sufficient for evaluating enhancer activity. Eleven (73%) of the 15 enhancers were located in the intergenic regions and four (27%) were in the introns of *KLF* genes (Additional file 1: Figure S3B). The higher frequency of enhancers in the intergenic regions is consistent with previous reports concerning enhancer distribution [3,48] and could also be explained by the fact that 83% DHSs were located beyond the proximal regions (>2 kb) in this study (Additional file 1: Figure S3A). The statistical analysis for enhancer distribution (Additional file 1: Figure S3B) indicated that enhancers tend to be distal [3]. Taken together, the present findings demonstrated that approximately two thirds of erythroid-specific or putative erythroid-specific DHSs were enhancers. Indeed, the majority of the DHSs were found to possess enhancer functions, supporting our original hypothesis that the high-throughput mRNA-seq and DHS mapping together provided a powerful mean for the identification of potential enhancers in the genome.

### More than half of erythroid-specific or putative erythroid-specific DHSs activate KLF promoters in erythroid cells

**Figure 4:**
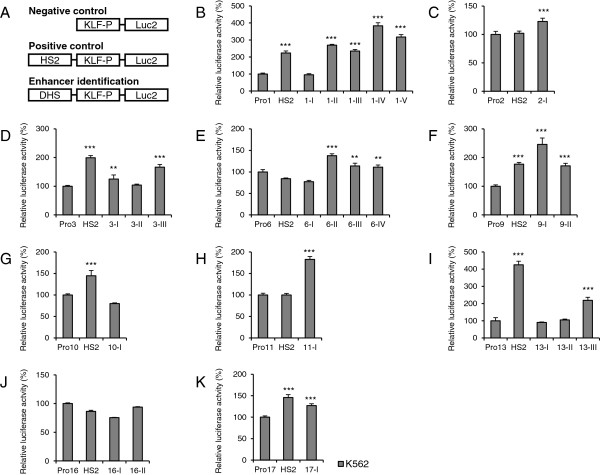
**Enhancer identification in K562 cells using the DLR assay with KLF-Ps. (A)** The constructs used in the reporter assay system are depicted. The pGL4.10 constructs containing respective KLF-Ps only were set as negative controls. The pGL4.10 constructs with KLF-Ps and enhancer HS2 were employed as positive references. The pGL4.10 constructs with DHSs under investigation in the place of HS2 upstream of KLF-Ps were used to evaluate the enhancer activities of DHSs. **(B-K)** Enhancer activities evaluation of the 23 DHSs in K562 cells. The firefly luciferase activity was normalized to that of *Renilla* luciferase. The relative luciferase activities of KLF-Ps–only constructs were normalized as 100%. DHSs with significantly (*p* < 0.01) higher activities than that of the negative control were defined as enhancers. Standard deviations were shown as error bars above each column. Asterisks indicate the statistically significant differences between the addition of DHSs and the KLF-Ps only in K562 cells ***p < 0.001, **p < 0.01.

**Figure 5:**
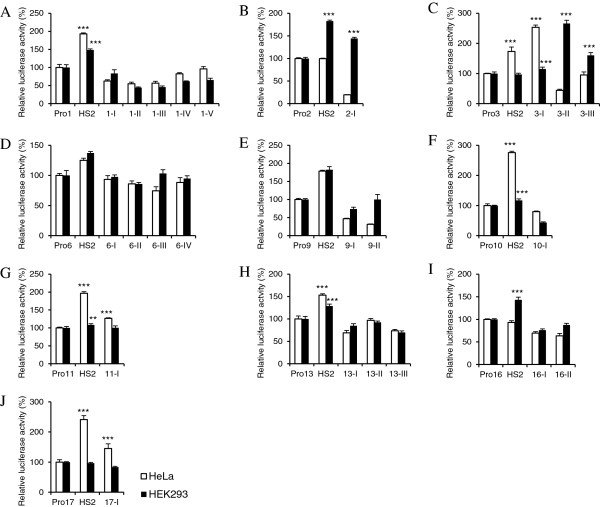
**Enhancer identification in non-erythroid HeLa and HEK293 cells using the DLR assay with *KLF*-Ps. (A-J)** Enhancer identification of 23 DHSs in HeLa (blank bar) and HEK293 (black bar) cells. The constructs used were the same as those shown in Figure 4A. DLR assay and data analysis were performed as described in Figure 4.

In different cells, enhancers activate gene expression by interacting with corresponding promoters. Therefore, we evaluated the enhancer activities of all the 23 DHSs in driving their respective KLF-Ps (Additional file 2: Table S2) in K562 cells. In total, 10 KLF-Ps were cloned and their activities were examined using the DLR assay (Additional file 2: Table S3). HS2 only activated some KLF-Ps in K562, HeLa, and HEK293 cells, and thus was used as a positive reference in this assay (Figures 4 and 5). DHSs that significantly (*p* < 0.01) increased the activities of corresponding KLF-Ps were considered as enhancers. Of the 23 DHSs, 15 (65%) displayed enhancer activities with respective KLF-Ps (Figure 4 and Table 1) in K562 cells. Importantly, some DHSs exhibited promoter specificity; for example, DHSs such as KLF6-I, KLF10-I, KLF13-II, and KLF16-I were strong enhancers on minP, but failed to activate their own promoters. In contrast, KLF3-I, KLF6-II, KLF6-III, and KLF13-III demonstrated strong enhancer activities with KLF3, KLF6 or KLF13 promoters, but not with minP, indicating that KLF3-I, KLF6-II, KLF6-III, and KLF13-III are gene-specific enhancers, and that their enhancer activities are independent of the TATA box [49] (Table 1). The distribution of erythroid KLF enhancers is shown in Additional file 1: Figure S3C.

**Table 1:** **Characteristics of erythroid-specific or putative erythroid-specific DHSs of ten human *KLF *genes**

**KLF**	**DHS**	**Cs**	**FAIRE**	**H3K4me1**	**H3K27ac**	**GATA-1**	**NF-E2**	**minP**	**KLF-P**		
			**K562**					**K562**	**K562**	**HeLa**	**HEK293**
**KLF1**	I	Y	Y	Y							
	II	Y	Y	Y*	Y*	Y		Y	Y		
	III	Y		Y*	Y*	Y		Y	Y		
	IV	Y	Y		Y*	Y		Y	Y		
	V		Y	Y*	Y*		Y	Y	Y		
**KLF2**	I	Y	Y	Y	Y	Y		Y	Y		Y
**KLF3**	I	Y	Y	Y	Y*				Y	Y	Y
	II	Y	Y	Y	Y*						Y
	III	Y	Y	Y	Y			Y	Y		Y
**KLF6**	I	Y	Y	Y	Y			Y			
	II			Y	Y				Y		
	III	Y		Y	Y*				Y		
	IV	Y		Y	Y*			Y	Y		
**KLF9**	I		Y	Y*	Y*	Y		Y	Y		
	II		Y	Y		Y		Y	Y		
**KLF10**	I			Y*				Y			
**KLF11**	I	Y	Y	Y	Y*			Y	Y	Y	
**KLF13**	I			Y*							
	II			Y*				Y			
	III			Y					Y		
**KLF16**	I		Y	Y*	Y*			Y			
	II	Y	Y	Y	Y						
**KLF17**	I	Y	Y	Y*	Y*			Y	Y	Y	

DHS conservation (Cs) was observed across 32 placental mammals, including humans, chimps, mice, and rabbits. The chromatin accessibility using FAIRE, enhancer-associated histone modifications (H3K4me1 and H3K27ac), and TFBSs for erythroid GATA-1 and NF-E2 on DHSs in erythroid K562 cells were extracted from UCSC Genome Browser. The enhancers characterized from the erythroid-specific or putative erythroid-specific DHSs were summarized in the last four columns. Y: yes. *: this histone modification is K562 specific or putative K562 specific.

### Approximately > 67% KLF enhancers are erythroid specific

To further investigate the erythroid specificity of KLF enhancers, we transiently transfected constructs with KLF-Ps into non-erythroid HeLa and HEK293 cells. DHSs that activated KLF-Ps in K562 cells but not in HeLa and HEK293 cells were considered as erythroid-specific KLF enhancers. DLR assay (Figure 5) revealed that 10 enhancers located in the genomic regions of KLF1-II, III, IV, and V; KLF6-II, III, and IV; KLF9-I and II; and KLF13-III were erythroid specific. Coincidentally, the mouse homologue of KLF1-II has been previously identified as an erythroid-specific enhancer [40]. These results provide strong evidence that the erythroid specificity of DHSs determines the erythroid specificity of enhancers. The distribution of these erythroid-specific KLF enhancers is shown in Additional file 1: Figure S3D.

### The nature of erythroid-specific KLF enhancers was validated by bioinformatic analyses

In general, enhancers are characterized by species conservation [10], characteristic H3K4me1 and H3K27ac enrichment [14], chromatin accessibility [DNase I hypersensitivity, Formaldehyde-Assisted Isolation of Regulatory Elements (FAIRE) sensitivity] [2,8,50], and binding capacity of TFs and coactivators [14]. According to the UCSC Genome Browser, of the 10 erythroid-specific KLF enhancers identified in this study, only five were conserved in placental mammals [51], whereas all the 10 enhancers were enriched with H3K4me1 and/or H3K27ac modifications [52], seven of which were erythroid specific, and six were occupied by erythroid-specific TFs GATA-1 and/or NF-E2 in erythroid K562 cells [53] (Table 1), further supporting their proposed roles as erythroid-specific enhancers.

**Figure 6:**
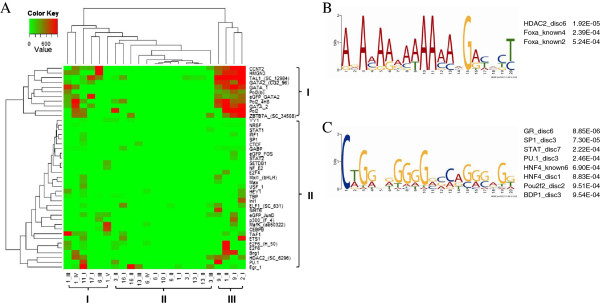
**Bioinformatic analyses of erythroid-specific KLF enhancers. (A)** Heat map of TFs binding to the 23 DHSs in K562 cells from ChIP-seq data after hierarchical clustering. **(B-C)** Motifs discovered from erythroid-specific KLF enhancers using MEME. The matched motifs with p-values < 0.001 are listed below corresponding figures.

The Txn Factor ChIP track synthesizes all the available ENCODE ChIP-seq data in different cell types; these data were used to build a full view of TF-binding sites (TFBSs) on the 23 DHSs. The signal strength of TF occupancy was quantified as a cluster score ranging from 0 to 1000. Because most (> 90%) TFBSs on the 23 DHSs occurred in K562 cells, we drew a heat map of cluster scores for this erythroid cell type after K-means clustering. As shown in Figure 6A, class II clustered four non enhancers (3-II, 16-II, 1-I, and 13-I), four minP-driving enhancers (16-I, 6-I, 10-I, and 13-II), three KLF P-driving enhancers (13-III, 6-II, and 3-I), and two minP- and KLF-P-dual driving enhancers (6-IV and 3-III). These 13 DHSs were bound by fewer TFs than the 10 DHSs in class I and III, which could account for their impaired or deficient enhancer activity in K562 cells. Of the remaining 10 DHSs, with the exception of 6-III, all were enhancers on both promoters in K562 cells because of the binding of multiple TFs. Furthermore, 11-I, 17-I, and 2-I were strong enhancers in K562 cells but were not erythroid specific because of the strong binding of TFs in other cell types. The remaining seven DHSs were erythroid-specific KLF enhancers. As shown in the vertical axis, TFs clustered to different classes: class I clustered several erythroid differentiation-related TFs (GATA-1, TAL1, and GATA-2) [22], and class II clustered enhancer-related factors (BATF, c-Fos, JunD, MEF2A, p300, PU.1, NFKB, STAT1, and SP1), histone modifiers and chromatin remodelers [HDAC2, p300, SETDB1, SIRT6, SWI/SNF components (Brg1 and Ini1), HMGN3, and Ini1] [12]. The features of TFs mentioned above were obtained from the NCBI Reference Sequence (RefSeq) database (http://www.ncbi.nlm.nih.gov/refseq/). In summary, in the open chromatin state, binding of TFs largely determines the enhancer activity of DHSs.

The ChIP-seq data of the UCSC Genome Browser did not cover all the TFBSs on the genome. Therefore, we performed de-novo motif analysis using the Multiple Em for Motif Elicitation (MEME) online software and annotated these motifs based on ENCODE-motifs by using TOMTOM [54]. ENCODE-motifs database covers all known motifs for each factor curated from TRANSFAC, Jaspar and Protein Binding Microarray (PBM) experiments and their enrichment within corresponding TF-binding experiments, as well as novel regulatory motifs discovered by systematic application of several motif discovery tools (including MEME, MDscan, Weeder, AlignACE) and evaluated based on their enrichment relative to control motifs within TF-bound regions (Kheradpour P, Kellis M: ENCODE-motifs: systematic analysis of regulatory motifs associated with transcription factor binding in the human genome, submitted). The conserved motifs among erythroid-specific KLF enhancers are shown in Figure 6B (E-value: 5.4 × 10^-4^) and Figure 6C (E-value: 2.6 × 10^1^); the p-values of their occurrence in DHSs were 1.03 × 10^-11^–1.78 × 10^-6^, and 4.91 × 10^-9^–3.14 × 10^-5^, respectively. Motif 1 (Figure 6B) could be the binding sites of histone modifier HDAC2 and enhancer-related TF foxa [55], and motif 2 (Figure 6C) could be the motifs of enhancer-related TFs SP1, FoxA2, PU.1 [12], and so on. The annotated motifs of HDAC2, SP1, STAT, and PU.1 were consistent with the TFBSs in cluster II of the heat map (Figure 6A).

### Erythroid-specificity of KLF enhancers is validated using the biochemical electrophoresis mobility shift assay

**Figure 7:**
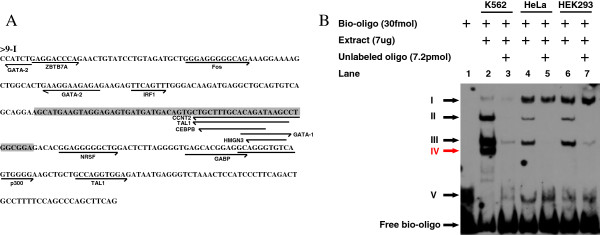
**Biochemical analysis of erythroid-specific KLF enhancers. (A)** Potential TFBSs on KLF9-I. TF occupancies on the erythroid-specific KLF enhancer KLF9-I were annotated using Txn Factor ChIP track and ENCODE-motifs on the UCSC Genome Browser. **(B)** Result of EMSA analysis. Biotin-labeled oligos were incubated with nuclear protein extracts of K562, HeLa, or HEK293 cells in the presence or absence of unlabeled competing oligos. Shifted bands are indicated by arrowheads on the left. Band IV (red) was found to be erythroid specific and was only detected in K562 cells, but not in HeLa or HEK293 cells.

Because the discovered motifs were mainly enhancer related, we performed the electrophoresis mobility shift assay (EMSA) to verify the erythroid specificity of erythroid-specific KLF enhancers. KLF9-I was selected as an example. Data from the UCSC Genome Browser indicate that sequence conservation of KLF9-I was observed in neither vertebrates nor placental mammals, but it was decorated with H3K4me1 and H3K27ac enhancer marks (Table 1) and harbored binding sites of erythroid-specific GATA-1 in erythroid K562 cells (Figure 7A). Because individual TFBSs can be relatively short and degenerate, they tend to be clustered to achieve precise temporal and developmental stage specificities [56]. Factors bound to these sequences often interact with common coactivators, which, in turn, recruit the basal transcription machinery [57,58]. We used EMSA to further investigate whether erythroid-specific TFs or cofactor complexes bind to KLF9-I *in vitro*. Putative TFBSs in KLF9-I were annotated using the Txn Factor ChIP track and ENCODE-motifs on UCSC Genome Browser (Figure 7A). Sequence analysis indicated that the motif of enhancer-related protein p300 [3,14,59] was embedded in KLF9-I (Figure 7A), which may account for the enhancer nature of KLF9-I, whereas the motif of GATA-1 may account for the erythroid specificity of KLF9-I. A pair of oligos (shadowed in Figure 7A) against KLF9-I were designed using the bioinformatic analysis. The EMSA result (Figure 7B) demonstrated that at least one protein complex in erythroid K562 cell extracts (band IV) specifically bound to the oligos, whereas no such binding was observed in non-erythroid HeLa and HEK293 cell extracts, implying that an erythroid-specific TF or cofactor complex bound to KLF9-I enhancer and drives KLF9 expression in erythroid cells. The other four universal bands suggested that KLF9-I may exert some universal functions in other cell types through recruitment of some basic transcriptional regulatory protein complexes. However, KLF9 has not been shown to function in erythroid cells. The detailed mechanisms by which KLF9-I mediates transcriptional regulation of KLF9 in erythroid cells need further investigation.

## Discussion

**Figure 8:**
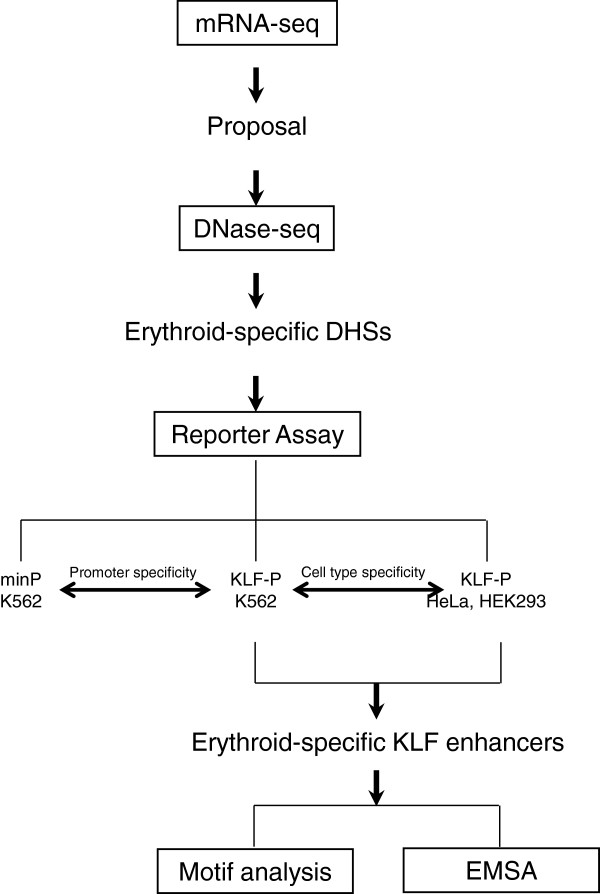
Methodology of identification and characterization of erythroid-specific KLF enhancers.

Mapping regulatory elements to the genes they regulate is of great importance to understand gene expression and functions. In this study, we provide a feasible strategy to extensively identify gene- and cell-specific enhancers from DHSs based on a rigorous and practical high-throughput sequencing technique (Figure 8). First, we refined the expression patterns of human KLFs from mRNA-seq data and proposed that the higher expression of eight KLFs in erythroid cells may be ascribed to the presence of erythroid-specific enhancers. Second, we screened erythroid-specific DHSs in the genomic regions of 17 human KLFs from DNase-seq dataset from four erythroid cell types and seven non-erythroid cell types, which largely improved the accuracy of prediction. Third, we extensively evaluated enhancer activities of all the 23 erythroid-specific or putative erythroid-specific DHSs using the DLR assay for promoter and cell-type specificities. Lastly, we validated the enhancer nature and erythroid specificity of erythroid-specific KLF enhancers through bioinformatic and biochemical analyses.

As the major contributors to cell- and developmental stage-specific gene expression, enhancers have been extensively predicted by many genome-wide approaches, including sequences conservation-, motif-, and chromatin-based computation methods [10,48,59]; ChIP-based analysis of TF binding such as CBP/p300 [13] or histone modifications (H3K4me1 and H3K27ac) [14]; or DHSs and FAIRE mapping [3,50]. The application of these approaches indicates that enhancers have unique properties that differentiate them from other CREs. However, enhancers predicted by these methods need to be validated further by using *in vitro* transient reporter gene system or *in vivo* transgenic systems. It is unrealistic to characterize all cell- and gene-specific enhancers within the whole human genome. In addition, these methods can not simultaneously take into account both cell and gene specificities of enhancers. In this study, using the next-generation high-throughput sequencing combined with multiple enhancer activity tests, we comprehensively characterized the enhancers confined to the genomic regions of a typical family of KLFs to explore both cell and gene specificities. The expression data in mRNA-seq may indicate the presence of enhancers, and DNase-seq data suggest the location of these enhancers. DHS mapping has higher positive rate than in-silico prediction methods and takes the advantage of unbiased property over ChIP-based methods, which only predict a subset of enhancers bound by one or several TFs and largely limited by the quality of antibodies. Reporter assays have the advantages of saving labor and time over animal models. With respect to the identification of erythroid-specific DHSs, we used cell lines originated from inner cell mass (HESC), ectoderm (HeLa), mesoderm (GM12878 and hTH2), and endoderm (HepG2 and CACO2) as control cell types for erythroid-specific DHSs screening. However, it is possible that certain erythroid-specific DHSs were excluded due to the presentation of similar DHSs as a result of binding by a different set of TFs in these non-erythroid cell lines. This setback may be remedied by the employment of high-resolution genome-wide *in vivo* footprinting [60]. Moreover, three primary erythroid cells were used for DHSs screening, which increased the validity of erythroid-specific DHSs. Although some subtle differences, but not specificity, were observed among DHSs at different developmental stages of erythroid cells, the similarities are actually unclear (Figure 2, Additional file 1: Figures S1 and S2). K562 cells are actually suitable for identifying erythroid-specific enhancers. However, there appears to be a limitation in characterizing the developmental stage specificity of enhancers using this cell line because it is at an early stage of erythroid differentiation. In the future, we wish to analyze this using appropriate erythroid cell lines at different developmental stages or animal models. In reporter assays, control cell lines HEK293 and HeLa are also representatives of different germ layers, mesoderm and ectoderm. Thus, the application of these non-erythroid control cell lines guaranteed the reliable and unbiased identification of cell-specific enhancers using our system. Beside cell-type specificity, we also explored gene specificity of enhancers, which is of great importance in gene expression and remains a challenge in this field. Furthermore, we validated the credibility of the identified erythroid-specific KLF enhancers through integrated bioinformatic and biochemical approaches to determine sequence conservation, TF binding, and histone modification markers. Identification of erythroid-specific KLF enhancers will facilitate the understanding of transcription, expression, and associated functions of *KLF* genes in erythroid cells and provide useful information to estimate the frequency of DHSs as gene- and cell-specific enhancers within the whole genome. With proper selection of cell types used as controls for DHS mapping and also employed for reporter assays, the approach described in this study should be applicable to a wide range of cells and genes of interest.

For example, a family of KLF members was selected to determine whether enhancers located in the genomic regions of KLFs were associated with their expression in erythroid cells. Here, we compared the expression of KLF mRNA transcripts in erythroid cells (ESER, FLER, and PBER) with those in non-erythroid cells (HESC) using mRNA-seq analysis. The present results reveal that eight KLFs (KLF1, KLF3, KLF6, KLF9, KLF10, KLF11, KLF13, and KLF16) show relatively higher expressions in erythroid cells than those in non-erythroid cells (Figure 1), which was proposed to be mainly determined by the presence of erythroid-specific CREs, particularly enhancers, embedded in DHSs of their genomic regions (Figure 2, Additional file 1: Figure S1, Additional file 2: Table S1). Ten erythroid-specific KLF enhancers were finally identified in the genomic regions of KLF1, KLF6, KLF9, and KLF13, indicating that these KLFs were erythroid-specific enhancers-driven genes and harbored potential biology in erythroid cells. However, despite their high expression in erythroid cells, no erythroid-specific enhancer was identified in the genomic regions of KLF3, KLF10, KLF11, and KLF16. The enhancers in the genomic regions of KLF3 and KLF11 were excluded because they demonstrated enhancer activities in non-erythroid cells as well (Figures 4 and 5, and Table 1). For KLF10 or KLF16, although the identified erythroid-specific or putative erythroid-specific DHSs in their genomic regions activated minP in K562 cells, no enhancer activating KLF-Ps was characterized, reflecting that DHSs identified in their genomic regions may work as promoter-specific enhancers. This is understandable because neither KLF10 nor KLF16 have been reported to be involved in erythroid differentiation and development. However, the lack of erythroid-specific enhancers in some KLFs with high erythroid expression may be caused by the limitation and bias of our arbitrary cut-offs of genomic regions. Some erythroid-specific KLF enhancers could exist beyond these confined regions (approximately 100 kb) or even on different chromosomes. These missed enhancers can be found by combining our system with the genome-wide chromosome conformation capture (3C)-base technology in the future, which may help in comprehensively understanding KLF transcription in erythroid cells. DHSs in the genomic region of *KLF2* and *KLF17* did not appear to show erythroid-specific enhancer activity, which may account for their abolished expressions in erythroid cells during mRNA-seq analysis (Figure 1A). In contrast, other KLFs (KLF4, KLF5, KLF7, KLF8, KLF12, and KLF15) were relatively highly expressed in non-erythroid cells (Figure 1), indicating that they were probably not erythroid-specific genes and that their genes expression could be driven by non-erythroid specific or universal CREs (Additional file 1: Figure S2A-E, G). No erythroid-specific or putative erythroid-specific DHSs were identified in the genomic region of *KLF14* (Additional file 1: Figure S2F), which could explain why its mRNA transcript could not be detected in cell types examined in this study.

In this study we identified ten novel erythroid-specific enhancers in the genomic regions of human KLFs (KLF1, KLF6, KLF9, and KLF13), of which KLF1-II, a homolog of murine EHS1, acted as an erythroid-specific enhancer [40]. Identification of erythroid-specific KLF enhancers may reveal novel mechanisms that regulate the transcription and functions of these KLFs in erythroid cells. Till date, KLF1, KLF6, and KLF13 have been identified to be critical regulators in erythroid cells [30,33,35,37]. KLF9 (BTEB1) is a broadly expressed TF with high expression in the developing brain, thymus, epithelia, smooth muscle of gut and bladder, vertebrae, and cartilage primordial and is implicated to play a role in the regulation of cell proliferation and differentiation [61]. *Klf9*^−/−^ mice have a normal lifespan, but impaired specific behavioral activities and decreased small intestinal villi [62,63]. *Klf9*^−/−^ female mice show uterine hypoplasia, reduced litter size, increased incidence of neonatal deaths in offspring and defects in parturition [64]. At present, the biology of KLF9 in erythroid cells has not been reported; thus, the identification of erythroid-specific KLF9 enhancers implies that KLF9 could be a novel KLF member that may play critical roles in erythroid cells. Future studies are warranted to investigate the functions of KLF9 in hematopoiesis, and the mechanisms by which the two identified erythroid-specific KLF9 enhancers regulate KLF9 gene transcription and expression in erythroid cells.

## Conclusions

The primary goal of this study is to develop a methodology to characterize enhancers from massive data generated by high-throughput sequencing technology. Using the high-throughput sequencing technique, we have provided a feasible and practical strategy to extensively identify gene- and cell-specific enhancers from DHSs. Application of our strategy led to the identification of ten erythroid-specific enhancers in the typical KLF family; their enhancer nature and erythroid specificity were confirmed using bioinformatic and biochemical analyses. Identification of erythroid-specific KLF enhancers indicates the relatively high expressions and some important functions of the corresponding KLFs in erythroid tissues.

## Methods

### Cell culture

K562, HeLa, and HEK293 cells were used for transient transfection to examine the enhancer activities in the DLR assay. K562 cells were cultured in RPMI1640 Medium (Gibco) with 10% fetal bovine serum (Hyclone) and penicillin (100 U/ml)-streptomycin (0.1 mg/ml) (Invitrogen), and HeLa and HEK293 cells were cultured in Dulbecco’s Modified Eagle Medium (Gibco) with 10% fetal bovine serum (Hyclone) and penicillin (100 U/ml)-streptomycin (0.1 mg/ml) (Invitrogen). All cells were maintained at 37 °C with 5% CO_2_ in a humidified incubator.

### Transcriptome sequencing and gene expression analysis

mRNA-seq was originally designed to explore the dynamic transcriptomes during human erythroid differentiation and development (Yang Y, Wang H, Chang KH, Qu H, Zhang Z, Xiong Q, Qi H, Cui P, Lin Q, Ruan X, *et al*: Transcriptome dynamics during human erythroid differentiation and development, submitted). In Brief, we extracted total RNA from HESC, ESER, FLER, and PBER, and depleted 18S and 28S ribosomal RNAs before constructing cDNA libraries. Next, we used the ABI SOLiD System to perform massively parallel ligation sequencing and mapped the sequence reads to human reference sequence [release Mar. 2006 (NCBI36/hg18)]. Gene expression intensity was calculated by normalizing the read counts to RPKM according to the gene length and total mapped reads, and genes with RPKM < 0.01 were removed.

### Quantitative real-time PCR

Total RNA was extracted from HESC, ESER, FLER, and PBER cells using TRIZOL® Reagent (Invitrogen, 15596–018) and DNA contamination was removed using the TURBO DNA-free™ Kit (Ambion, AM1907). DNA-free RNA was reverse transcribed using the RevertAid First Strand cDNA Synthesis Kit (Thermo Scientific, K1622) according to manufacturer’s instruction. Primers were designed using Primer 5 (Additional file 2: Table S4). PCR were performed in triplicate using Maxima® SYBR Green/ROX qPCR Master Mixes (2×) (Fermentas, K0223) and CFX96™ Real-Time PCR Detection System (Bio-rad), and data were analyzed using the CFX Manager™ Software. The KLF transcript levels were first calculated by referring to those of 18S ribosomal RNA, and the expression levels of KLFs in ESER, FLER, and PBER were normalized to those in HESC. The statistical significance of differences between individual KLFs’ expressions in erythroid cell types and those in HESC were calculated using the independent-samples *t*-test.

### Digital DNase I sequencing and erythroid-specific or putative erythroid-specific DHS selection

In this study, the DNase-seq data used were obtained from the University of Washington [41,60], and are available through the UCSC Genome Browser (http://genome.ucsc.edu) and the NCBI Gene Expression Omnibus (GEO) data repository under accessions GSE29692 and GSE32970. DHSs were identified using an algorithm developed by the University of Washington [65]. In this study, FDR threshold of 0.5% was used to define DHS for each cell type. The domains of KLF loci were defined as extensions from 70 kb upstream of TSSs to 20 kb downstream of the poly (A) sites. ESER, FLER, and PBER cells represent primary erythroid cells at different developmental stages, and K562 cells represent erythroleukemia cells. In DHS screening, all other cell types were employed as non-erythroid control cell types. DHSs in these domains were considered to be erythroid specific if they were only present in erythroid cells and were identified as putative erythroid specific if they were present in erythroid cells and exhibited much lower peaks in one or two non-erythroid cell types.

### DNA manipulation

To generate firefly luciferase reporter constructs with minP, the identified 23 erythroid-specific or putative erythroid-specific DHSs were amplified from human blood genomic DNA with Pfu DNA Polymerase (Promega, M7741) and inserted upstream of minP in the pGL4.23 expression vector (Promega, E8411). To further generate firefly luciferase reporter constructs with KLF-Ps, TSSs of individual KLFs were predicted from UCSC Genome Browser, and fragments of approximately 1 kb in length upstream of TSSs (Additional file 2: Table S2) were amplified and cloned into pGL4.10 vector, followed by inserting DHSs upstream of the corresponding KLF-Ps. The activities of KLF-Ps were examined and used as baselines. HS2, a classical enhancer in β-globin LCR, was cloned into the corresponding vectors upstream of minP or KLF-Ps and used as positive controls in the DLR assay [16]. The primers used in this study are listed in Additional file 2: Tables S5 and S6. The integrity of the reporter constructs was confirmed using restriction digestions and sequencing.

### Transient transfection and DLR assay

Cells were seeded into 48-well plates. K562 cells (1.5 × 10^5^/well) were transiently transfected with 500 ng of firefly luciferase vector and 0.75 ng of a *Renilla* luciferase vector, pRL-TK (Promega, E2441), using Lipofectamine LTX and Plus Reagent (Invitrogen, 15338–100). HeLa (7 × 10^4^/well) and HEK293 (7 × 10^4^/well) cells were similarly transfected using the Lipofectamine 2000 Transfection Reagent (Invitrogen, 11668–019) as per the manufacturer’s instructions. Forty-eight hours after transfection, cells were harvested to prepare for the cell lysates, and luciferase activities were immediately measured with the Dual-Luciferase Reporter Assay System (Promega, E1910) as per the manufacturer’s instructions. Transient transfections were repeated at least twice, and every construct was transfected in triplicates. Standard deviations were shown as error bars above respective columns. For data processing, firefly luciferase activity was normalized to that of *Renilla* luciferase in all the groups, and the relative activity of each promoter was normalized as 1. The statistical significance of differences between promoters and DHSs were analyzed using one-way ANOVA function in R language.

### Bioinformatic analyses

Data of conserved elements in placental mammals [51], layered H3K4me1 and layered H3K27ac [52], and Txn Factor ChIP data [53] in the regions of 23 DHSs were obtained from UCSC Genome Browser and summarized in Table 1.

TFs binding to the 23 DHSs were collected from Txn Factor ChIP track on UCSC Genome Browser. A heat map was drawn after K-means clustering using R language.

Conserved motifs embedded in erythroid-specific KLF enhancers were analyzed using the MEME software (http://meme.ebi.edu.au/meme/cgi-bin/meme.cgi), and these de novo discovered motifs were searched against the ENCODE-motifs database (http://www.broadinstitute.org/~pouyak/motif-disc/human/) using TOMTOM algorithm (http://meme.ebi.edu.au/meme/cgi-bin/tomtom.cgi) [54].

### EMSA

Bio-11-dUTP (Ambion, AM8450) and TdT (New England Biolabs, M0315s) were used to label the 3′-OH of single-stranded oligos (5′-AGC ATG AAG TAG GAG AGT GAT GAT GAC AGT GCT GCT TTG CAC AGA TAA GCC TGG CGG A-3′, 5′-TCC GCC AGG CTT ATC TGT GCA AAG CAG CAC TGT CAT CAT CAC TCT CCT ACT TCA TGC T-3′) and complementary oligos were annealed as per the manufacturer’s instructions. Nuclear proteins of K562, HeLa, and HEK293 cells were extracted using the rapid micro-preparation method (lysis buffer: 10 mM Hepes [pH7.9], 10 mM KCl, 1.5 mM MgCl_2_, 0.5 mM PMSF, 0.5 mM DTT; high-salt extraction buffer: 20 mM Hepes [pH7.9], 25% glycerol, 0.42 M NaCl, 1.5 mM MgCl_2_, 0.2 mM EDTA, 0.5 mM PMSF, 0.5 mM DTT) [66]. Protein concentrations were measured using the BCA Protein Assay Kit (Pierce, 23225). EMSA was performed in 20 μl reaction mixture, containing 30 fmol biotin-labeled oligos and 7 μg nuclear extract with or without 7.2 pmol unlabeled oligos using the LightShift Chemiluminescent EMSA Kit (Pierce, 20148) according to the manufacturer’s instruction (Figure 7B).

### Availability of supporting data

DNase-seq data are available through the UCSC Genome Browser (http://genome.ucsc.edu/cgi-bin/hgFileUi?db=hg19&g=wgEncodeUwDnase), or through the NCBI Gene Expression Omnibus (GEO) data repository (accession numbers: GSE29692, GSE32970). The other data sets supporting the results of this article are included within the article and the additional files.

## Abbreviations

DHS: DNase I hypersensitive site; CRE: *Cis*-regulatory element; KLF: Krüppel-like factor; DLR: Dual-luciferase reporter; minP: Minimal promoter; KLF-P: KLF promoter; ChIP: Chromatin immunoprecipitation; LCR: Locus control region; ENCODE: ENCyclopedia of DNA Elements; TF: Transcription factor; HESC: Human embryonic stem cells; ESER: Embryonic stem cells-derived erythroid cells; FLER: Fetal liver-derived erythroid cells; PBER: Adult mobilized peripheral blood CD34+ cells derived erythroid cells; FDR: False discovery rate; TSS: Transcription start site; FAIRE: Formaldehyde-assisted isolation of regulatory elements; TFBS: TF binding site; MEME: Multiple Em for Motif Elicitation; EMSA: Electrophoresis mobility shift assay; RPKM: Reads per kilobase of exon model per million mapped reads.

## Competing interests

The authors declare no competing interests.

## Authors’ contributions

QX and ZZ carried out molecular genetic studies, DLR assay, EMSA and wrote the paper; KHC performed cell culture and wrote the paper; YL and XR performed cell culture; HQ, RS, PJS and JAS performed high-throughput DNase I profiling and analyzed data; HW and YY performed high-throughput transcriptome sequencing; YY analyzed mRNA-seq data and performed the bioinformatic and statistical analyses; HQ and YL performed RT-PCR and DLR assay; GS and QL helped to draft the manuscript; JAS and XF conceived of the study, and participated in its design and coordination and wrote the paper. All authors read and approved the final manuscript.

## Supplementary Material

Additional file 1: Figure S1 Chromatin profiles of *KLF* genes containing erythroid-specific (arrow and column in green) or putative erythroid-specific (arrow and column in blue) DHSs. *KLF* loci were arbitrarily defined as extension from 70 kb upstream of the TSSs to 20 kb downstream of the poly (A) sites. Erythroid-specific or putative erythroid-specific DHSs were respectively marked with green and blue arrowheads and named with Roman numbers. **Figure S2.** Chromatin profiles of *KLF* genes without erythroid-specific or putative erythroid-specific DHSs. **Figure S3.** Distribution statistics of the identified erythroid-specific or putative erythroid-specific DHSs and enhancers in the genomic regions of KLFs. A. Statistics of the distribution of identified erythroid-specific or putative erythroid-specific DHSs relative to *KLF* genes and TSSs. In total, 18 (78%) and five (22%) DHSs are localized to the intergenic and intronic regions, respectively; 15 (65.2%) DHSs are located far distal (>10 kb) to TSSs, four (17.4%) DHSs are located distal (2-10 kb) to TSSs, and four (17.4%) DHSs are located in proximal (<2 kb) promoter regions. DHS KLF1-III contains TSS (Additional file 1: Table S1). B. Statistics of the distribution of the identified enhancers under the control of minP in K562 cells relative to *KLF* genes and TSSs. C. Statistics of the identified erythroid KLF enhancer distribution relative to the *KLF* genes and TSSs. D. Statistics of the identified erythroid-specific KLF enhancer distribution relative to *KLF* genes and TSSs. Click here for file

Additional file 2: Table S1 The relative position and length of 23 identified erythroid-specific or putative erythroid-specific DHSs. **Table S2.** Position, length, GC content, chromatin accessibility (DNase I hypersensitivity), and references of ten KLF promoters used in enhancer assays.** Table S3.** Activities of KLF promoters measured by the luciferase reporter assay. **Table S4.** Primers used for measuring the expression patterns of KLFs in real-time PCR. **Table S5.** Primers used for amplification of erythroid-specific DHS fragments inserted in luciferase reporter constructs. **Table S6.** Primers used for amplification of KLF promoters. **Supplementary References.** Click here for file
