# Supplementary material for: Comprehensive characterization of erythroid-specific enhancers in the genomic regions of human Krüppel-like factors
Source: BMC Genomics. 2013 Aug 28;14:587. doi: 10.1186/1471-2164-14-587 (PMC3846580; doi:10.1186/1471-2164-14-587)

Supplementary Figure S1

A

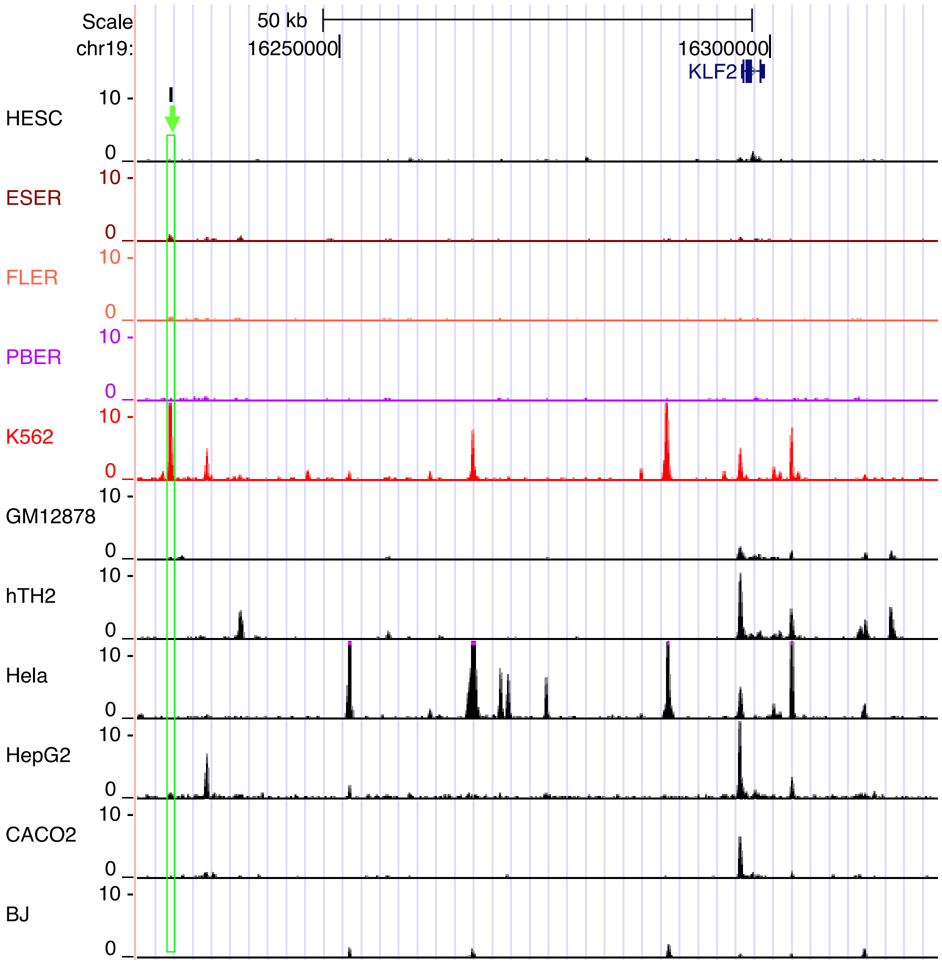

B

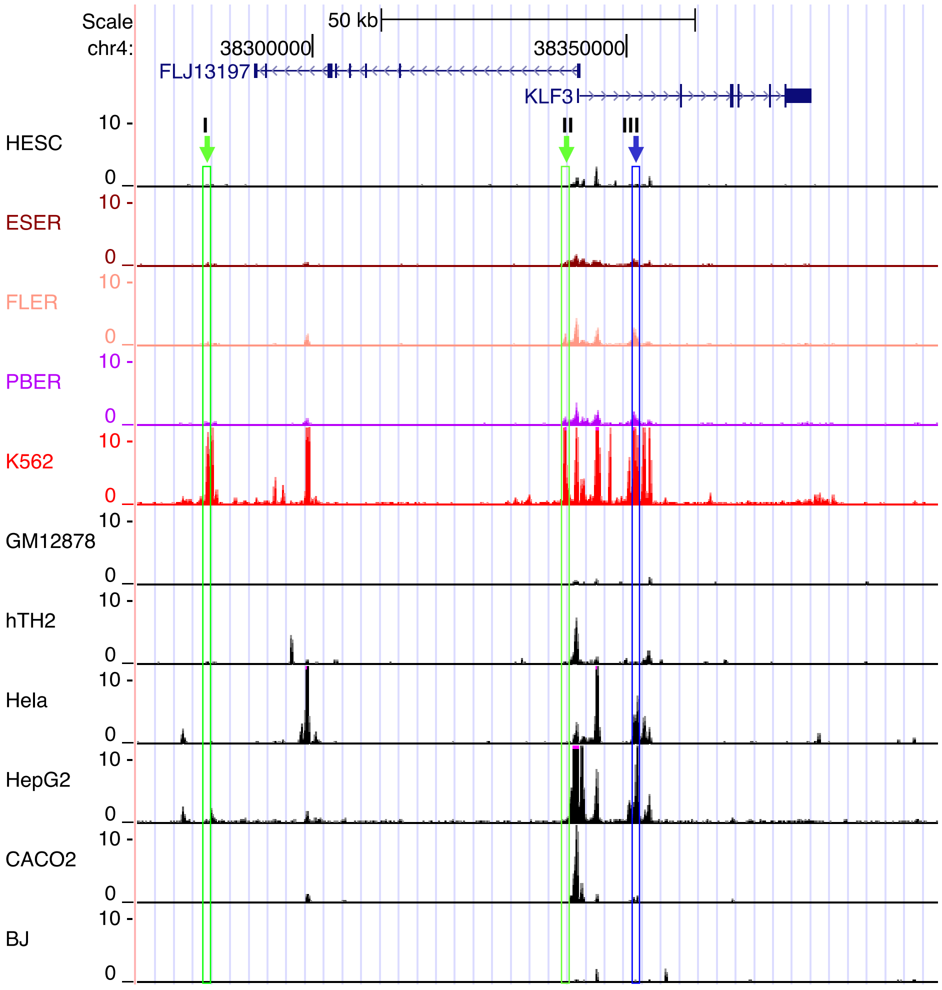

Supplementary Figure S1

C

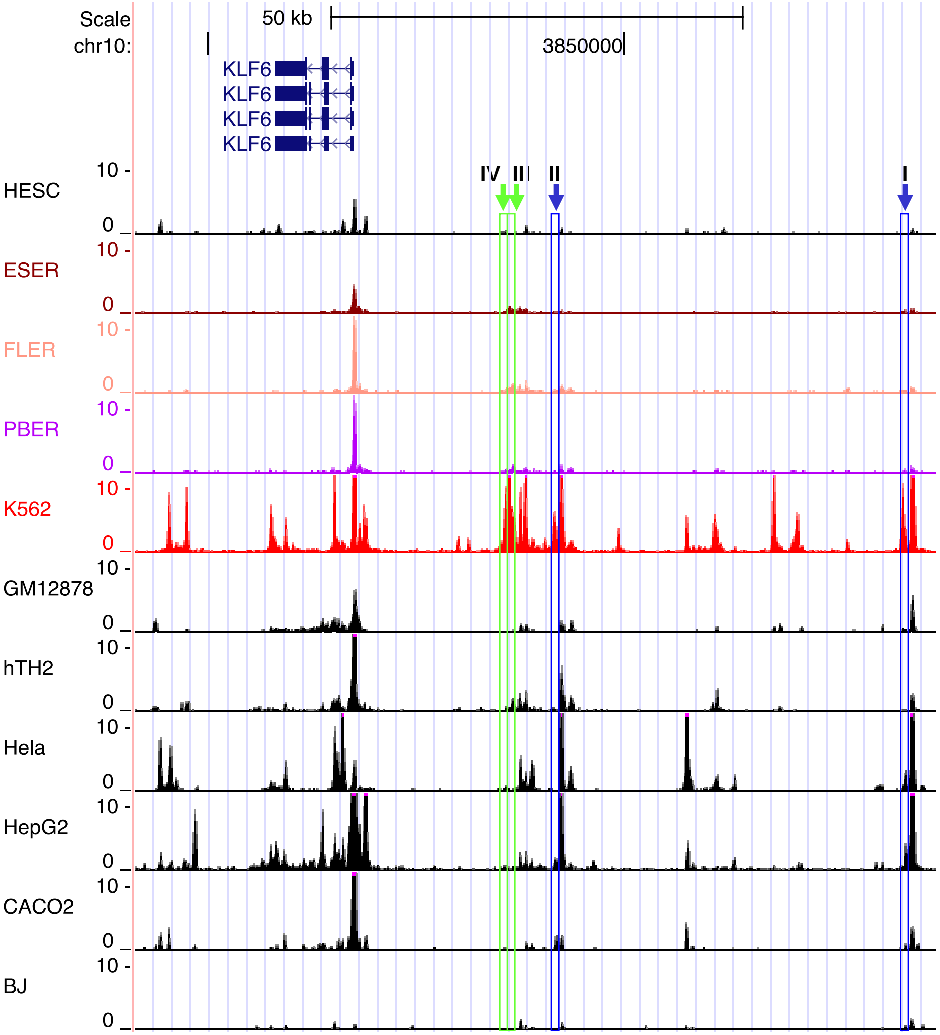

D

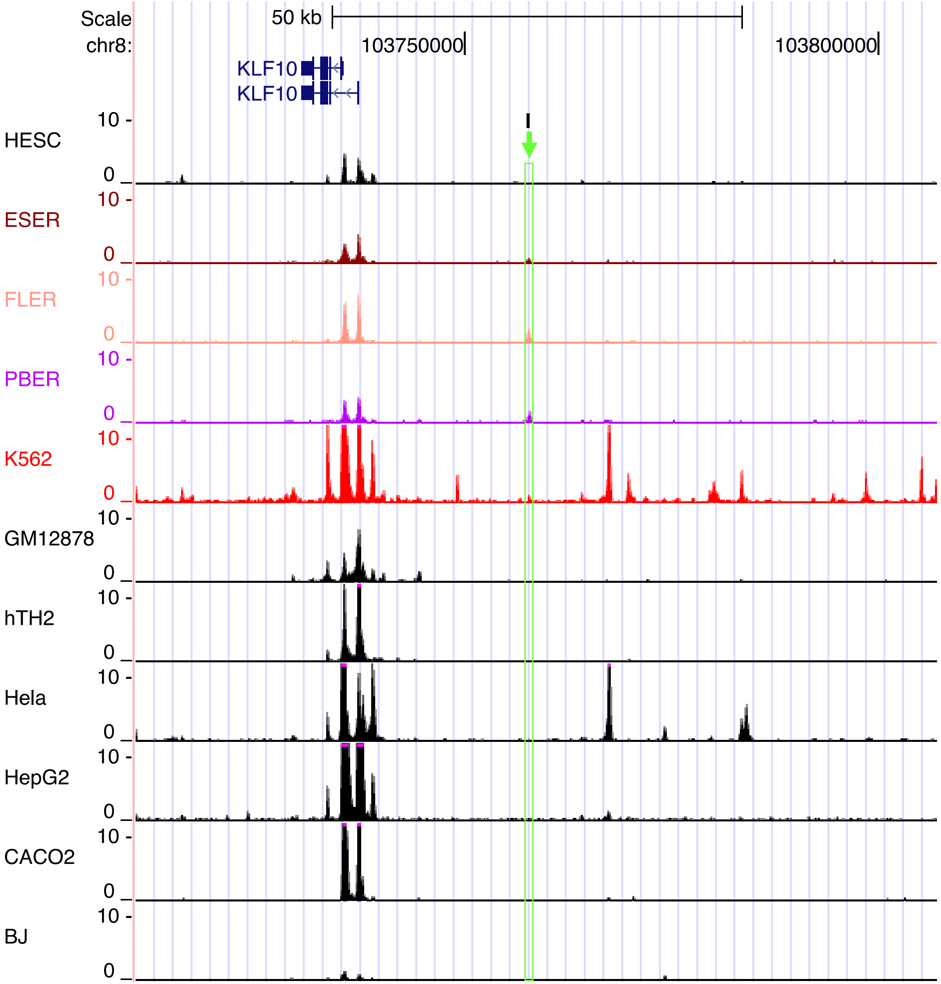

Supplementary Figure S1

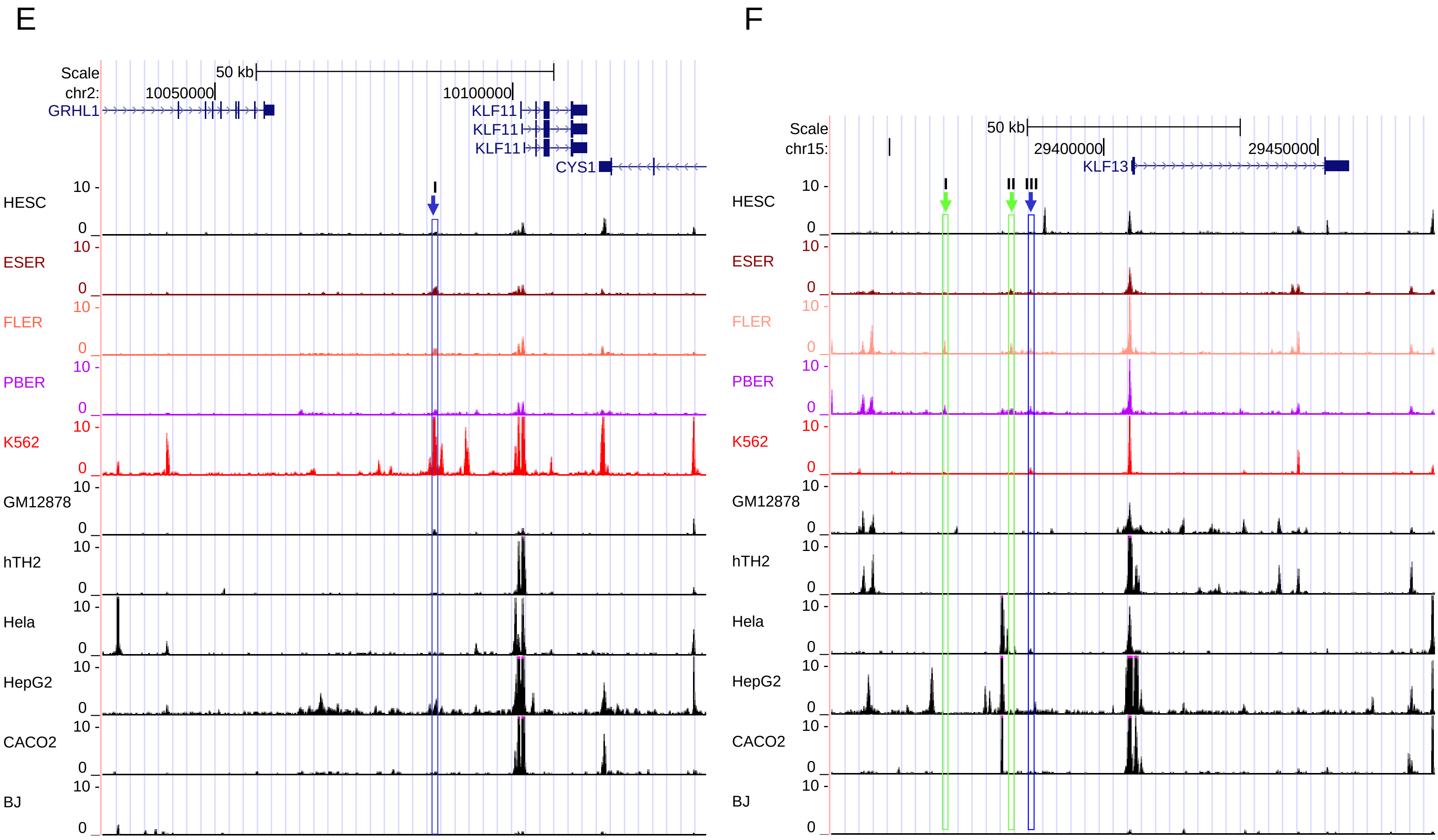

Supplementary Figure S1

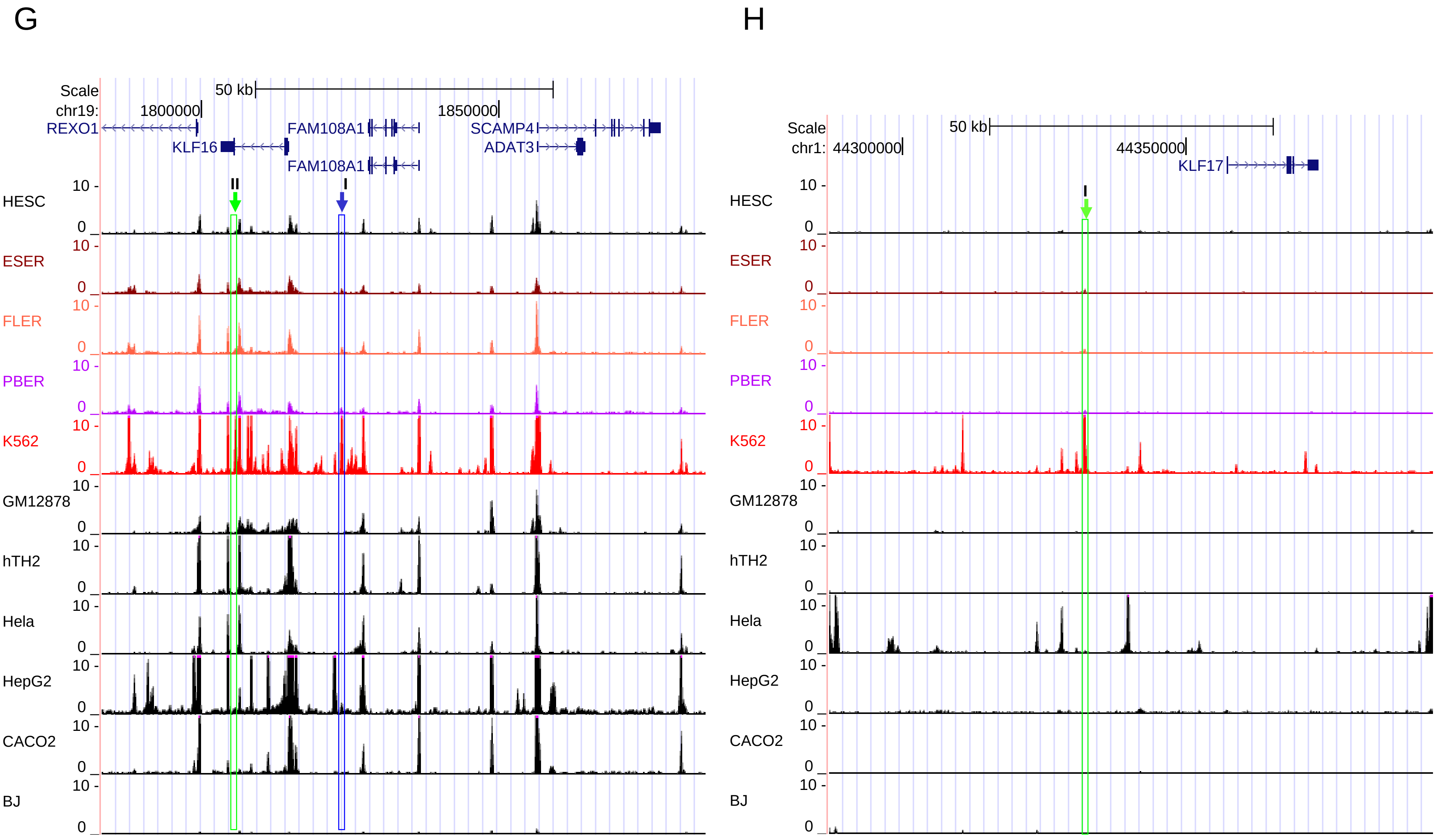

Supplementary Figure S2

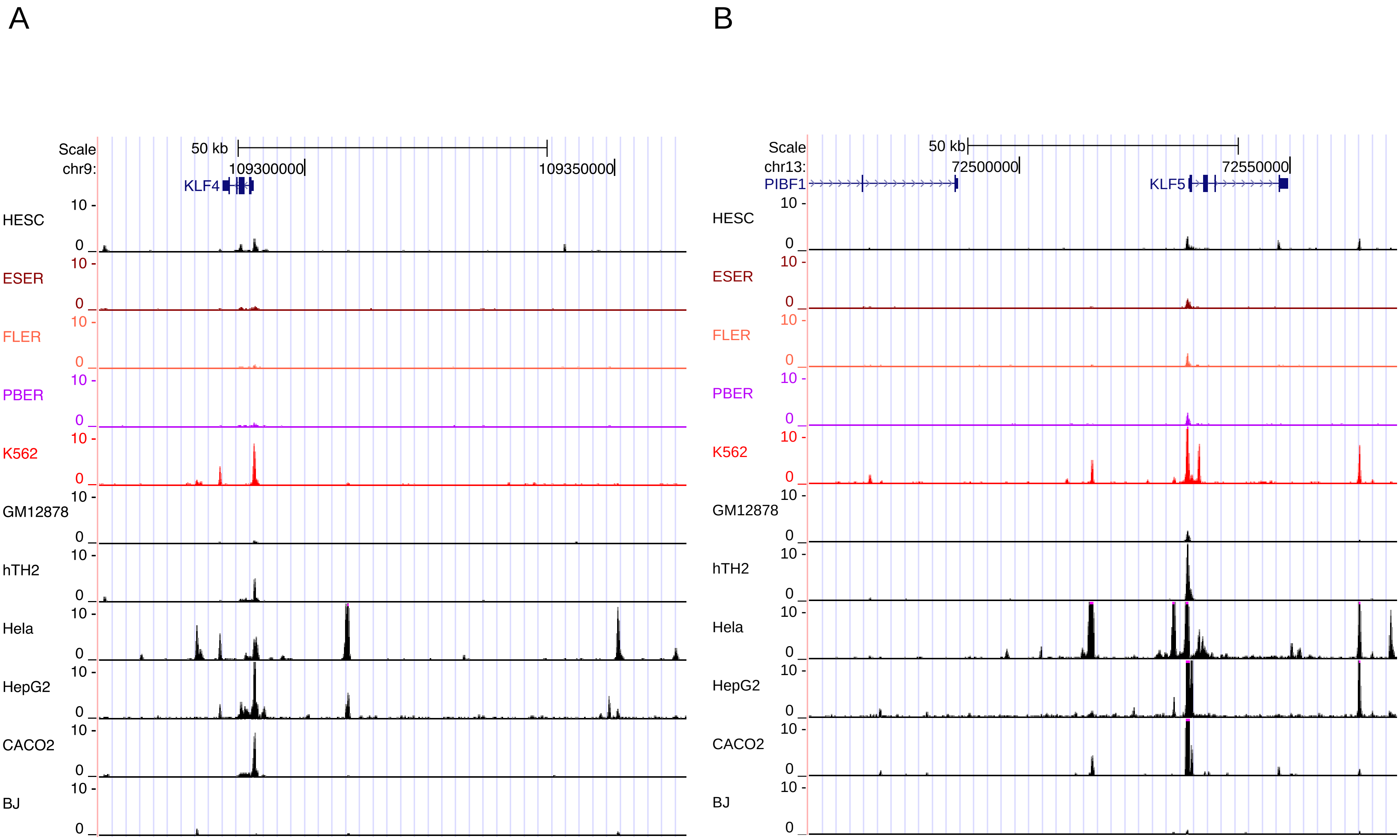

Supplementary Figure S2

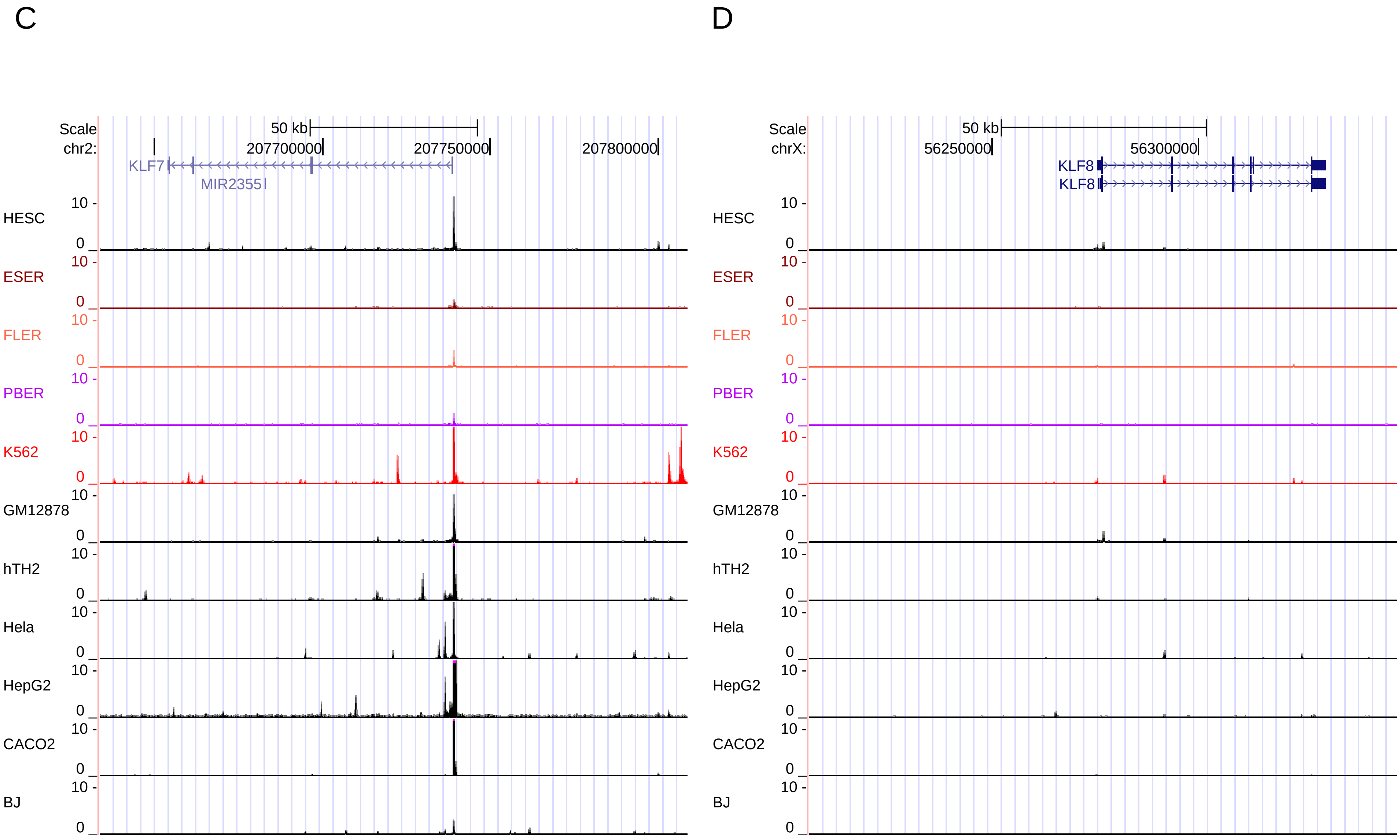

Supplementary Figure S2

E

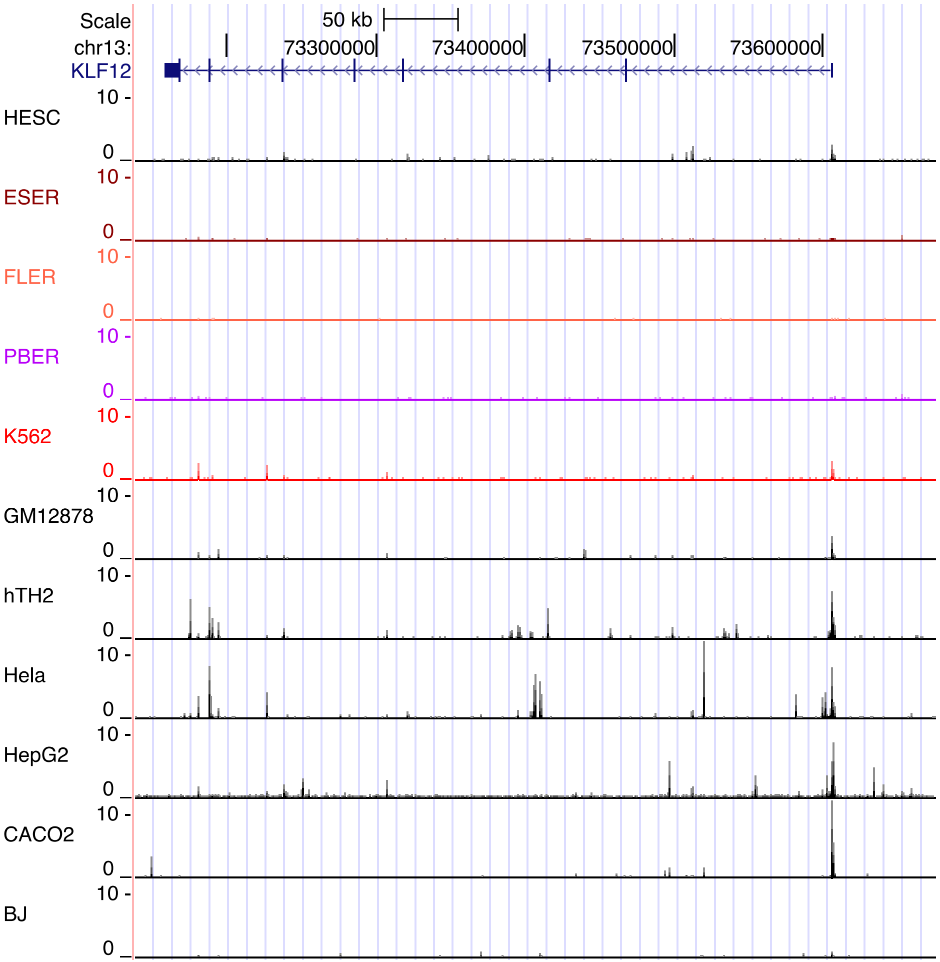

F

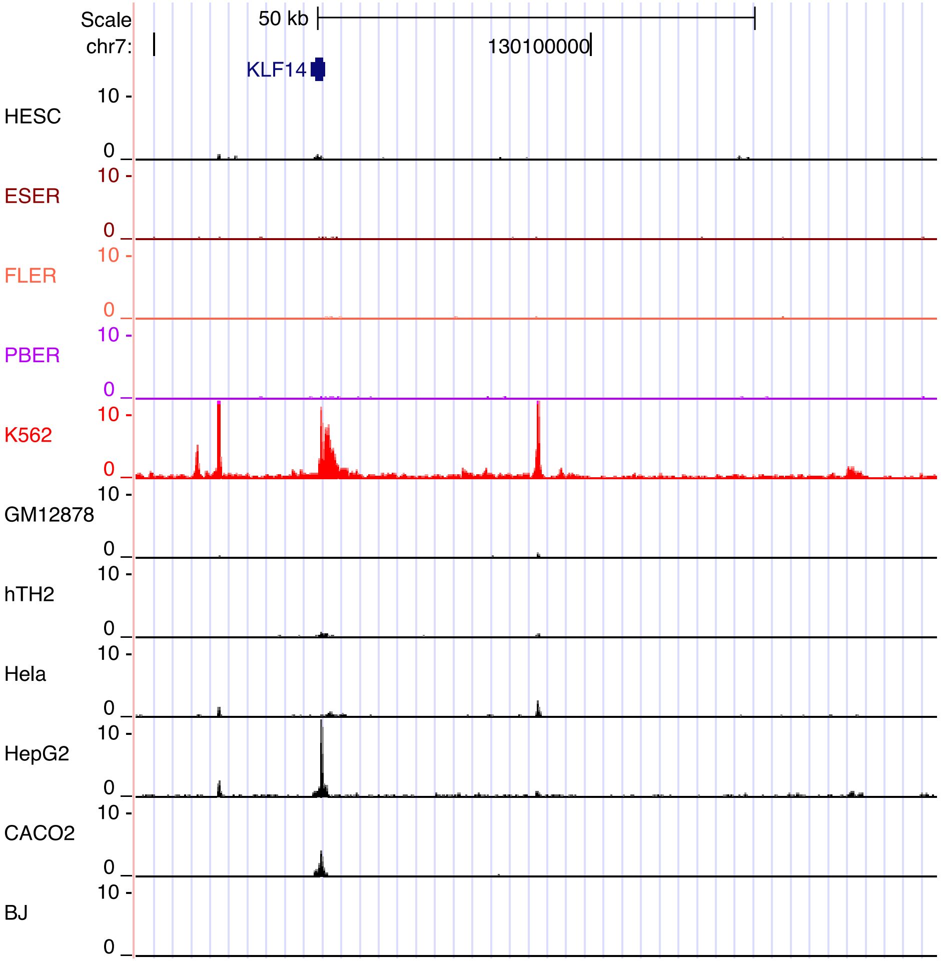

Supplementary Figure S2

G

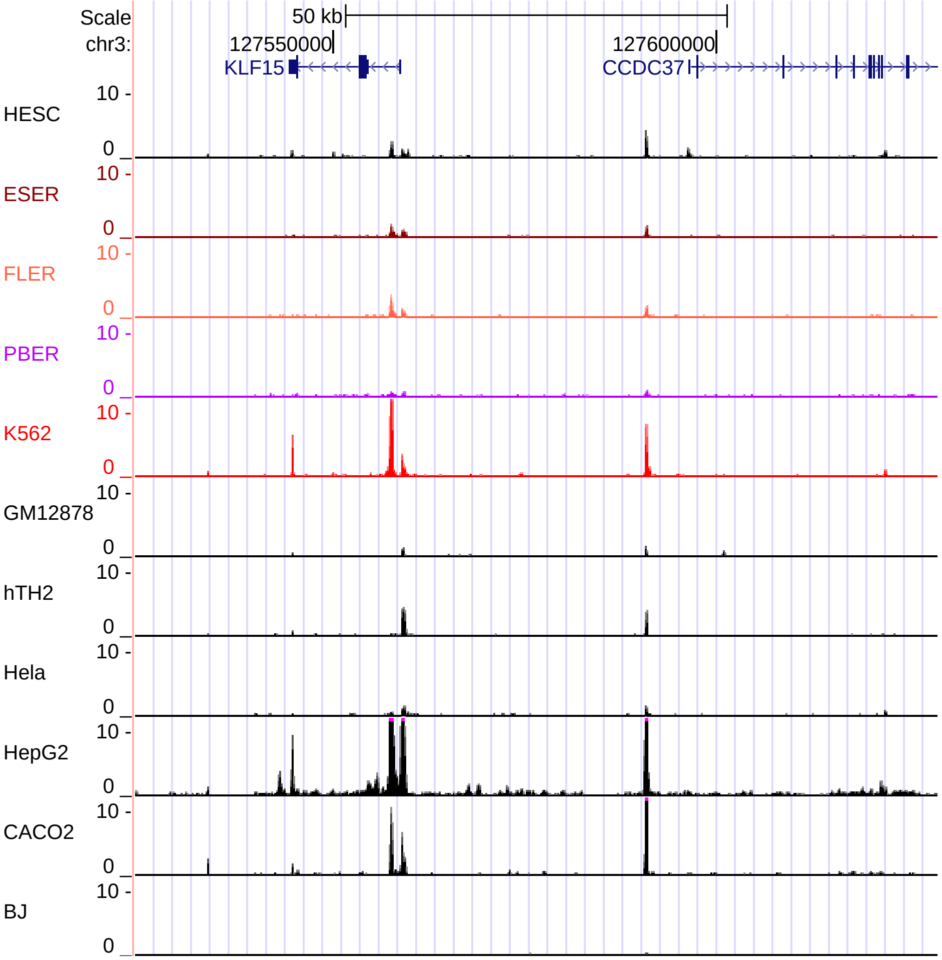

Supplementary Figure S3

A Distribution of erythroid DHSs

B Distribution of enhancers under minP in K562 cells

C Distribution of erythroid KLF enhancers

D Distribution of erythroid-specific KLF enhancers

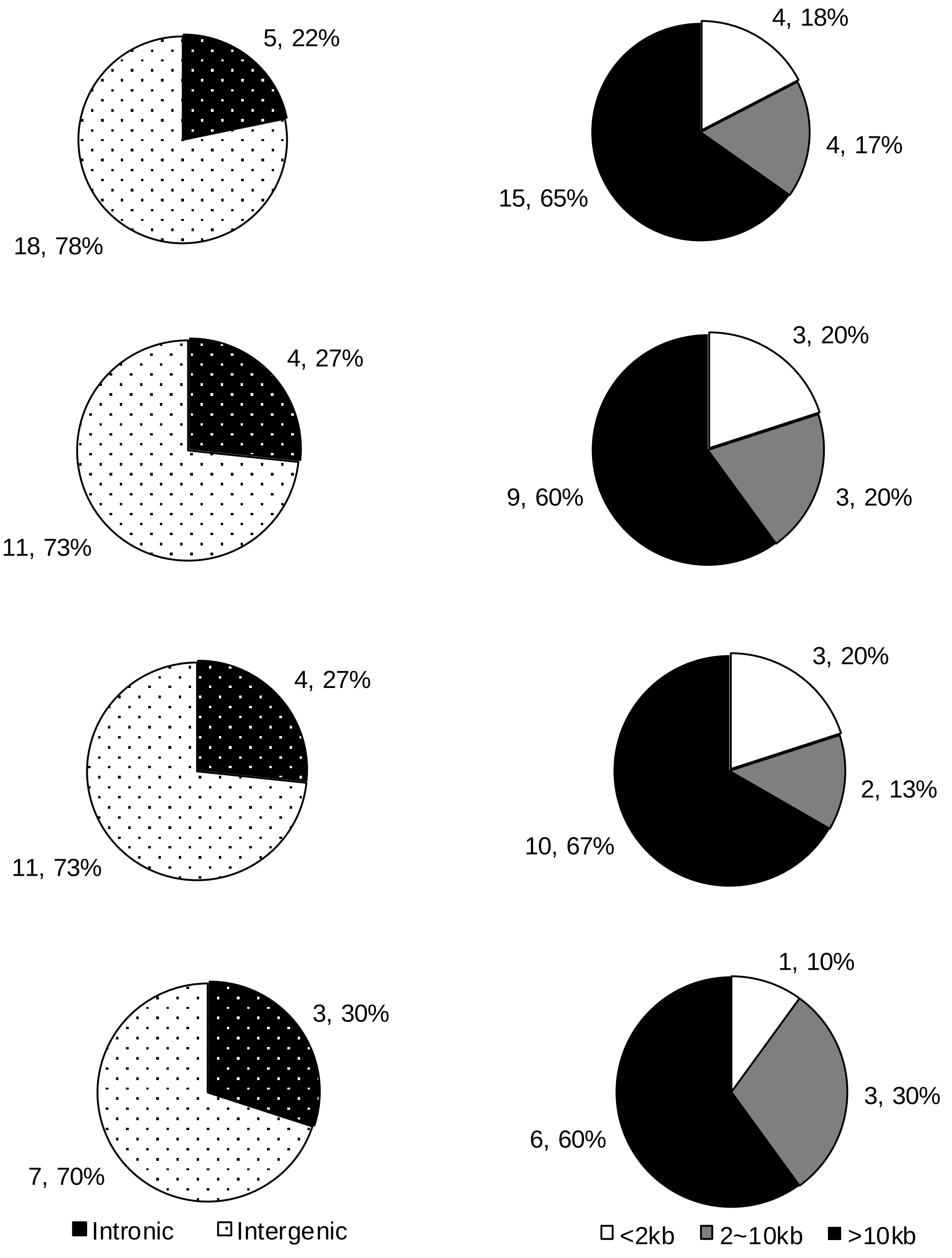

Supplement: Additional file 1: Figure S1 — Chromatin profiles of KLF genes containing erythroid-specific (arrow and column in green) or putative erythroid-specific (arrow and column in blue) DHSs. KLF loci were arbitrarily defined as extension from 70 kb upstream of the TSSs to 20 kb downstream of the poly (A) sites. Erythroid-specific or putative erythroid-specific DHSs were respectively marked with green and blue arrowheads and named with Roman numbers. Figure S2. Chromatin profiles of KLF genes without erythroid-specific or putative erythroid-specific DHSs. Figure S3. Distribution statistics of the identified erythroid-specific or putative erythroid-specific DHSs and enhancers in the genomic regions of KLFs. A. Statistics of the distribution of identified erythroid-specific or putative erythroid-specific DHSs relative to KLF genes and TSSs. In total, 18 (78%) and five (22%) DHSs are localized to the intergenic and intronic regions, respectively; 15 (65.2%) DHSs are located far distal (>10 kb) to TSSs, four (17.4%) DHSs are located distal (2-10 kb) to TSSs, and four (17.4%) DHSs are located in proximal (<2 kb) promoter regions. DHS KLF1-III contains TSS (Additional file 1: Table S1). B. Statistics of the distribution of the identified enhancers under the control of minP in K562 cells relative to KLF genes and TSSs. C. Statistics of the identified erythroid KLF enhancer distribution relative to the KLF genes and TSSs. D. Statistics of the identified erythroid-specific KLF enhancer distribution relative to KLF genes and TSSs. [file 1471-2164-14-587-S1.pdf]
