# Supplementary material for: Comprehensive characterization of erythroid-specific enhancers in the genomic regions of human Krüppel-like factors
Source: BMC Genomics. 2013 Aug 28;14:587. doi: 10.1186/1471-2164-14-587 (PMC3846580; doi:10.1186/1471-2164-14-587)
Supplement: Additional file 2: Table S1 — The relative position and length of 23 identified erythroid-specific or putative erythroid-specific DHSs. Table S2. Position, length, GC content, chromatin accessibility (DNase I hypersensitivity), and references of ten KLF promoters used in enhancer assays. Table S3. Activities of KLF promoters measured by the luciferase reporter assay. Table S4. Primers used for measuring the expression patterns of KLFs in real-time PCR. Table S5. Primers used for amplification of erythroid-specific DHS fragments inserted in luciferase reporter constructs. Table S6. Primers used for amplification of KLF promoters. Supplementary References. [file 1471-2164-14-587-S2.doc]

# Supplementary tables

**Table S1 - T**he relative position and length of 23 identified erythroid-specific or putative erythroid-specific DHSs

| **DHS** | **Position^a^** | | **Length** |  | **DHS** | **Position^a^** | | **Length** |
| --- | --- | --- | --- | --- | --- | --- | --- | --- |
| **1-I E** | Upstream | -61112~-60783 | 330 |  | **6-IV E** | Upstream | -18469~-18161 | 309 |
| **1-II E** | Upstream | -637~-302 | 336 |  | **9-I PE** | Upstream | -71024~-70715 | 310 |
| **1-III E** | Promoter | -190~+147 | 338 |  | **9-II PE** | Intron I | +16193~+16495 | 303 |
| **1-IV E** | Intron I | +711~+972 | 262 |  | **10-I E** | Upstream | -20753~-20444^b^ | 310 |
| **1-V PE** | Downstream | +3145~+3402 | 258 |  | **11-I PE** | Upstream | -14446~-14124 | 323 |
| **2-I E** | Upstream | -66354~-66021 | 334 |  | **13-I E** | Upstream | -43731~-43420 | 312 |
| **3-I E** | Upstream | -58917~-58621 | 297 |  | **13-II E** | Upstream | -28087~-27773 | 315 |
| **3-II E** | Upstream | -2102~-1781 | 322 |  | **13-III PE** | Upstream | -23688~-23388 | 301 |
| **3-III PE** | Intron I | +8975~+9276 | 302 |  | **16-I PE** | Upstream | -9205~-8884 | 322 |
| **6-I E** | Upstream | -66260~-65904 | 357 |  | **16-II E** | Intron I | +8575~+8933 | 359 |
| **6-II PE** | Upstream | -24457~-24132 | 326 |  | **17-I E** | Upstream | -25165~-24844 | 322 |
| **6-III E** | Upstream | -18877~-18566 | 312 |  |  |  |  |  |

Note: *a: position relative to the TSS. *b: position relative to the TSS of KLF10 isoform a. E stands for erythroid specific while PE stands for putative erythroid specific

**Table S2 - Position, length, GC content, chromatin accessibility (DNase I hypersensitivity) and references of ten KLF promoters used in the enhancer assays**

| **Promoter** | **Position^a^** | **Length (bp)** | **GC content (%)** | **DNase I HS** | **References** |
| --- | --- | --- | --- | --- | --- |
| KLF1 | -950~+62 | 1013 | 58.1 | yes |  |
| KLF2 | -701~+131 | 833 | 70.3 | yes |  |
| KLF3 | -510~+292 | 803 | 79.1 | yes |  |
| KLF6 | -683~+248 | 932 | 72.1 | yes |  |
| KLF9 | +149~+951 | 803 | 57.9 | yes |  |
| KLF10 | -591~+143 | 735 | 72.7 | yes |  |
| KLF11 | -745~+152 | 898 | 73.2 | yes |  |
| KLF13 | -920~+134 | 1055 | 76.2 | yes |  |
| KLF16 | -1107~+97 | 1205 | 78.7 | yes |  |
| KLF17 | -1039~--52 | 988 | 51.0 | yes |  |

Note: *a: position relative to the TSS.

**Table S3 -** Activities of KLF promoters measured by the luciferase reporter assay

| **Promoter** | **Activities** | | |
| --- | --- | --- | --- |
|  | **K562** | **Hela** | **HEK293** |
| **minP** | 0.67^*^ |  |  |
| **KLF1** | 5.41 | 26.31 | 6.4 |
| **KLF2** | 98.41 | 1630.6 | 277.52 |
| **KLF3** | 5.95 | 1.69 | 1.71 |
| **KLF6** | 94.72 | 129.98 | 92.3 |
| **KLF9** | 10.61 | 24.94 | 5.7 |
| **KLF10** | 74.83 | 134.55 | 1.12 |
| **KLF11** | 16.46 | 4.83 | 2.43 |
| **KLF13** | 7.54 | 106.63 | 13.59 |
| **KLF16** | 143.64 | 1003.93 | 178.86 |
| **KLF17** | 0.24 | 20.08 | 5.45 |

Note: ^*^The numbers indicates the expression activity ratio of firefly luciferase to *Renilla* luciferase.

**Table S4 - Primers used for measuring the expression patterns of KLFs in real-time PCR**

| **DHS** | **Primers** | |
| --- | --- | --- |
|  | **Forward** | **Reverse** |
| **KLF1** | TTG CGG CAA GAG CTA CAC C | GTC AGA GCG CGA AAA AGC AC |
| **KLF2** | CTA CAC CAA GAG TTC GCA TCT | CCG TGT GCT TTC GGT AGT G |
| **KLF3** | AAG CTC CCA CTT GAA AGC ACT | AGG GCA AGA TGG TCA GAA CG |
| **KLF4** | TTC CCA TCT CAA GGC ACA CC | CAT GTG TAA GGC GAG GTG GT |
| **KLF5** | AGC TCA CCT GAG GAC TCA CA | TTC ATA TGC AGG GCC AGG TG |
| **KLF6** | CTG CCG TCT CTG GAG GAG T | TCC ACA GAT CTT CCT GGC TGT C |
| **KLF7** | GGC AGT GGA CAT CTT GCT CT | CCA CGG CAG AGA GAG TTT GT |
| **KLF8** | GCA GCC ATT ACA GTC CCA CT | TGC TGT AGT CCC TGC AGA CT |
| **KLF9** | ACA GTG GCT GTG GGA AAG TC | AAC TGC TTT TCC CCA GTG TG |
| **KLF10** | CTT CCG GGA ACA CCT GAT TTT | GCA ATG TGA GGT TTG GCA GTA |
| **KLF11** | CCC ATC TTC GCA CTC ACA CA | CAT GCT TCG TCA GGT GGT CA |
| **KLF12** | CAA CAC TAT TGT CGT GCC GC | GAA AGG GCC GTA GAT CCA GT |
| **KLF13** | AGA GGA AGC ACA AGT GCC A | TGA ACT TCT TCT CGC CCG TG |
| **KLF14** | GAG GTC TGT CAC ACC TGC TG | ACG CGT AAA CTT CTT GTC GCA |
| **KLF15** | CTG GCT GCA GCA AGA TGT AC | TCT TCT CGC ACA CAG GAC AC |
| **KLF16** | GAC TGC GCC AAA GCC TAC TA | TGA AGC GCT TGG AGC ACA GA |
| **KLF17** | GGA GTG CAC ACC TCT TGG AA | GCA GTA GCT CAT ACC ACG CT |
| **18S rRNA** | CAGCCACCCGAGATTGAGCA | TAGTAGCGACGGGCGGTGTG |

**Table S5 - Primers used for amplification of erythroid-specific DHS fragments inserted in luciferase reporter constructs**

| **DHS** | **Primers** | |
| --- | --- | --- |
|  | **Forward** | **Reverse** |
| **1-I** | GGG **GCTAGC** TTT GTG GTC GTC ATG GTG A | GGG **CTCGAG** ATG GGC TCT TGT CCT CTC T |
| **1-II** | GGG **GGTACC** CAA GGG TCC CCA GTA GAC | GGG **GCTAGC** GTA TTC TGG GAA GAA GCT G |
| **1-III** | GGG **GGTACC** CCC CAA CCC TTG ATA TTT GAC | GGG **GAGCTC** TGA GGA AGT CAT CCT GTG TG |
| **1-IV** | GGG **GGTACC** GGA TGT TCT GGG GGA AAA C | GGG **GAGCTC** GCT TTG GAA AGG GGT CTT G |
| **1-V** | GGG **GGTACC** TCC AGC TAA ATG GGA GAT GGG | GGG **GAGCTC** AAC CTA ACG CTG GCC TTT TTA G |
| **2-I** | GGG **GGTACC** GGA GAA CCC ACC ACC TGT | GGG **GCTAGC** CGC TCA GCT CCT GAG CT |
| **3-I** | CCC **GGTACC** TTT TTC TGG TAG CAA GTC T | CCC **GAGCTC** AGG GTA CTA AGC TGG GA |
| **3-II** | TTC ACT GAG CTA CCC AGC ACT | CTG GCT CAT AGT AGG TGC TCA |
| **3-III** | GGG **GGTACC** AGG AGC AGA GGA ATG ATC AC | GGG **GAGCTC** CTA AGC CCT CAC AAT TCT CC |
| **6-I** | GGG **GGTACC** GAC AGA GTG AGA TCC TGT | GGG **GAGCTC** TGG TAT CAT GTG AAA AAG TTA |
| **6-II** | GGG **GGTACC** GGG CAG GAA GTG AGC C | GGG **GAGCTC** TGG GGG ATT AAC ACC TTG |
| **6-III** | GGG **GGTACC** GCG TGC AAC TCT GTC GAT AAC | GGG **GAGCTC** AGC ACA TAT GCA CCA CAA GCC |
| **6-IV** | GGG **GGTACC** GAC AGA TTC CGT CAA TTT G | GGG **GAGCTC** AAT GCA GCA TGT GCT TAG G |
| **9-I** | GGG **GGTACC** CCA TCT GAG GAC CCA GAA C | GGG **GAGCTC** CTG AAG CTG GGC TGG AAA AG |
| **9-II** | GGG **GGTACC** GAG GTC TGC TGG AAC TGC | GGG **GAGCTC** CCT CTC ACT GAA TCC TGC |
| **10-I** | GGG **GGTACC** CAC CCA CCA ACA ATG TGA CTT G | GGG **GAGCTC** TTC CTC TCT GGG CTT TAC CAC |
| **11-I** | GGG **GGTACC** ACG TGG GCT GTT TTA TCT G | GGG **GAGCTC** GGG TGT CTC AGT CCC TG |
| **13-1** | GGG **GGTACC** ATG GAG TGA AAG GCA CAG AG | GGG **GAGCTC** AGC GGT TGT CAA GGG TTT C |
| **13-II** | GGG **GGTACC** CTG CAC AGA AGT GTG GCC | GGG **GAGCTC** TCC CCA CAG ACA AGG AGT C |
| **13-III** | GGG **GGTACC** CAC CCC CTT GAA TCT CAG | GGG **GAGCTC** CTT GTT TGG GGT GTG GTC |
| **16-I** | GGG **GGTACC** ACC GAG AGA AGT AGC CGA G | GGG **GCTAGC** GCA CTC TGC TAA CAG CCA C |
| **16-II** | CCC **GGTACC** AGC GCT TGG AGC ACA GA | CCC **GCTAGC** AGA GCT GGT AGG AAA TAA GA |
| **17-I** | GGG **GGTACC** GCA CTC ATC TTT ATA CGA | GGG **GCTAGC** CTC ACG TAG TTC AGA CT |

Note: The enzyme restriction sites are shown in bold.

**Table S6 - Primers used for amplification of KLF promoters**

| **Promoter** | **Primers** | |
| --- | --- | --- |
|  | **Forward** | **Reverse** |
| **KLF1** | GGG **AGATCT** GTC TTC CAA TGA GTG ACT ATG | GGG **CCATGG** GGC TGG CTG GTG CCC AC |
| **KLF2** | CCC **AGATCT** TGA ACT CCT GGC CTC GAG TGA | GCT GGC GAA AGT GGA GAA GGA |
| **KLF3** | GGG **AAGCTT** TGT GTG TTT TGG AAG CAG CG | GGG **CCATGG** TTT GCG AGG CGC GGT GCA |
| **KLF6** | GCT **AGATCT** AGG GAA ACA GAG CGG CGC AGA A | GCT **CCATGG** GTC GGG CCG GGT TGG ACG GA |
| **KLF9** | GGG **AGATCT** TTT CAC TAC AGC TCT TCG CGT | GGG **CCATGG** GGC GGT CGC AAG TTT ATT CG |
| **KLF10** | TTT **AGATCT** AAA TGC AGC TCA GTC GGT CAC C | AGA GGC ACC GAA GTT GAG CAT G |
| **KLF11** | GGG **AGATCT** TCT GGG TGA CTG CCC GTT TC | GGG **CCATGG** CGG CCG GGA GCA ACA AAG C |
| **KLF13** | GGG **AGATCT** CTG GCA CGT AGT TCG CTT CT | GGG **CCATGG** GCA CCG AAG AGT GAG CGC A |
| **KLF16** | GGG **AGATCT** TGC CTG GGA ATG GAA AGA G | GTA ATC CAC GCA CGC CAC |
| **KLF17** | CCC **AGATCT** TCT AAC TGG TGT CTC TGC A | GGG **CCATGG** CTA TTT ACA GCT GCG CCA A |

Note: The enzyme restriction sites are shown in bold.

# Supplementary references

1. Crossley M, Tsang AP, Bieker JJ, Orkin SH: **Regulation of the erythroid Kruppel-like factor (EKLF) gene promoter by the erythroid transcription factor GATA-1**. *J Biol Chem* 1994, **269**(22):15440-15444.

2. Chen X, Reitman M, Bieker JJ: **Chromatin structure and transcriptional control elements of the erythroid Kruppel-like factor (EKLF) gene**. *J Biol Chem* 1998, **273**(39):25031-25040.

3. Fabre S, Carrette F, Chen J, Lang V, Semichon M, Denoyelle C, Lazar V, Cagnard N, Dubart-Kupperschmitt A, Mangeney M *et al*: **FOXO1 regulates L-Selectin and a network of human T cell homing molecules downstream of phosphatidylinositol 3-kinase**. *J Immunol* 2008, **181**(5):2980-2989.

4. Funnell AP, Maloney CA, Thompson LJ, Keys J, Tallack M, Perkins AC, Crossley M: **Erythroid Kruppel-like factor directly activates the basic Kruppel-like factor gene in erythroid cells**. *Mol Cell Biol* 2007, **27**(7):2777-2790.

5. Gehrau RC, D'Astolfo DS, Prieto C, Bocco JL, Koritschoner NP: **Genomic organization and functional analysis of the gene encoding the Kruppel-like transcription factor KLF6**. *Biochim Biophys Acta* 2005, **1730**(2):137-146.

6. Engelmann D, Knoll S, Ewerth D, Steder M, Stoll A, Putzer BM: **Functional interplay between E2F1 and chemotherapeutic drugs defines immediate E2F1 target genes crucial for cancer cell death**. *Cell Mol Life Sci* 2010, **67**(6):931-948.

7. Gutierrez-Aguilar R, Froguel P, Hamid YH, Benmezroua Y, Jorgensen T, Borch-Johnsen K, Hansen T, Pedersen O, Neve B: **Genetic analysis of Kruppel-like zinc finger 11 variants in 5864 Danish individuals: potential effect on insulin resistance and modified signal transducer and activator of transcription-3 binding by promoter variant -1659G>C**. *J Clin Endocrinol Metab* 2008, **93**(8):3128-3135.

8. Kuroda E, Horikawa Y, Enya M, Oda N, Suzuki E, Iizuka K, Takeda J: **Identification of minimal promoter and genetic variants of Kruppel-like factor 11 gene and association analysis with type 2 diabetes in Japanese**. *Endocrine journal* 2009, **56**(2):275-286.

9. Mitsuma A, Asano H, Kinoshita T, Murate T, Saito H, Stamatoyannopoulos G, Naoe T: **Transcriptional regulation of FKLF-2 (KLF13) gene in erythroid cells**. *Biochim Biophys Acta* 2005, **1727**(2):125-133.
